# OOBO: A New Metaheuristic Algorithm for Solving Optimization Problems

**DOI:** 10.3390/biomimetics8060468

**Published:** 2023-10-01

**Authors:** Mohammad Dehghani, Eva Trojovská, Pavel Trojovský, Om Parkash Malik

**Affiliations:** 1 Department of Mathematics, Faculty of Science, University of Hradec Králové, 50003 Hradec Králové, Czech Republic; eva.trojovska@uhk.cz (E.T.); pavel.trojovsky@uhk.cz (P.T.); 2 Department of Electrical and Software Engineering, University of Calgary, Calgary, AB T2N 1N4, Canada; maliko@ucalgary.ca

**Keywords:** metaheuristic algorithm, one-to-one correspondence, exploration, exploitation, sensors, engineering

## Abstract

This study proposes the One-to-One-Based Optimizer (OOBO), a new optimization technique for solving optimization problems in various scientific areas. The key idea in designing the suggested OOBO is to effectively use the knowledge of all members in the process of updating the algorithm population while preventing the algorithm from relying on specific members of the population. We use a one-to-one correspondence between the two sets of population members and the members selected as guides to increase the involvement of all population members in the update process. Each population member is chosen just once as a guide and is only utilized to update another member of the population in this one-to-one interaction. The proposed OOBO’s performance in optimization is evaluated with fifty-two objective functions, encompassing unimodal, high-dimensional multimodal, and fixed-dimensional multimodal types, and the CEC 2017 test suite. The optimization results highlight the remarkable capacity of OOBO to strike a balance between exploration and exploitation within the problem-solving space during the search process. The quality of the optimization results achieved using the proposed OOBO is evaluated by comparing them to eight well-known algorithms. The simulation findings show that OOBO outperforms the other algorithms in addressing optimization problems and can give more acceptable quasi-optimal solutions. Also, the implementation of OOBO in six engineering problems shows the effectiveness of the proposed approach in solving real-world optimization applications.

## 1. Introduction

The term “optimization” refers to obtaining the optimal solution out of all available solutions to a problem [1]. Optimization appears widely in real-world issues. For example, the goal of engineers is to design a product with the best performance, traders seek to maximize profits from their transactions, and investors try to minimize investment risk, etc. [2]. These types of problems must be modeled mathematically and then optimized using the appropriate method. Each optimization problem is composed of three parts: (a) decision variables, (b) constraints, and (c) objective functions that can be modeled using Equations (1)–(4).

(1) $$\mathrm{Minimize}/\mathrm{Maximize}:\;f\left(x\right),\;x=\left[x_{1},x_{2},\;\ldots ,x_{m}\right]$$

Subject to:

(2) $$g_{i}\left(x\right)<0,\;\;i=1,2,\;\ldots ,\;p,$$

(3) $$h_{k}\left(x\right)=0,\;\;k=1,2,\;\ldots ,q,$$

(4) $$lb_{j}\leq x_{j}\leq ub_{j},\;\;j=1,2,\;\ldots ,m,$$

where m is the number of problem variables, $x=(x_{1},x_{2},\ldots ,x_{m})$ is the vector of problem variables, f(x) is the values of the objective function for problem variables, $g_{i}$ is the ith inequality constraint, p is the total number of inequality constraints, $h_{k}$ is the kth equality constraint, q is the total number of equality constraints, and $lb_{j}$ and $ub_{j}$ are the lower and upper bounds of the jth problem variable $x_{j}$, respectively.

Problem-solving techniques in the study of optimization problems fall into two categories. The first category consists of “exact algorithms” that find optimal solutions to these problems and guarantee the optimality of these solutions. The second category consists of “approximate algorithms,” which are usually designed to solve optimization problems that exact methods are unable to solve [3]. In contrast to exact algorithms, approximate algorithms are able to generate appropriate quality solutions for many optimization problems in a reasonable period of time. However, the important issue with approximate algorithms is that there is no assurance that the problem’s global optimal solution will be found [4]. As a result, solutions derived from approximation approaches are referred to as quasi-optimal [5]. A quasi-optimal solution should be as near to the global optimum as feasible.

Random-based optimization algorithms are among the most extensively utilized approximate algorithms in the solution of optimization problems. Optimization algorithms can give acceptable quasi-optimal solutions for objective functions by employing random operators and random scanning of the optimization problem’s search space [6]. The proximity of their offered quasi-optimal solution to the global optimum is the key criterion of optimization algorithms’ superiority over one another. Scholars created numerous optimization techniques in this respect with the goal of finding quasi-optimal solutions that are closer to the global optimum. These random-based optimization algorithms are used in solving combinatorial optimization problems.

The main question that arises is whether there is still a need to design new optimizers, given that numerous optimization algorithms have been produced. According to the No Free Lunch (NFL) theorem [7], even if an optimization method is very good at solving a certain set of optimization problems, there is no guarantee that it will be an effective optimizer for other optimization problems. As a result, it is impossible to declare that a specific method is the best optimizer for all optimization challenges. The NFL theorem has prompted researchers to design new optimizers to handle optimization issues in a variety of fields [8]. This motivated the authors of this study to develop a novel optimization approach to optimizing real-world engineering problems that are both effective and gradient-free.

The novelty and innovation of this paper are in developing a novel population-based optimization method called the One-to-One-Based Optimizer (OOBO) to handle diverse optimization problems. The main contributions of this paper are as follows:

- The key idea behind the suggested OOBO algorithm is the effective use of different members of the population and not relying on specific members during the population updating process.
- The suggested OOBO algorithm’s theory is discussed, and its mathematical model for applications in solving optimization problems is offered.
- OOBO’s ability to provide appropriate solutions is evaluated with fifty-two distinct objective functions.
- The effectiveness of OOBO in solving real-world applications is tested on four engineering design problems.
- The performance of OOBO is compared with eight well-known algorithms to assess its quality and ability.

The proposed OOBO approach has advantages, such as simple concepts, simple equations, and convenient implementation. The main advantage of OOBO is that it does not have any control parameters; therefore, the proposed approach does not need to adjust the parameters (of course, it should be mentioned, except for the population size, i.e., N and the maximum number of iterations of the algorithm, i.e., T, which are present in all metaheuristic algorithms due to the nature of population-based metaheuristic algorithms). In addition, the optimization process in the proposed OOBO ensures that a member of the population is employed solely to guide a member of the population in each iteration of the algorithm. Therefore, all members participate in guiding the OOBO population.

The rest of the paper is as follows: A literature review is presented in Section 2. Section 3 introduces the suggested OOBO algorithm. Section 4 contains simulation studies and results. The evaluation of OOBO for optimizing four real-life problems is presented in Section 5. Finally, conclusions and several recommendations for further research are stated in Section 6.

## 2. Literature Review

Optimization algorithms are classified into five types, based on their primary design concepts: (a) swarm-based, (b) physics-based, (c) evolutionary-based, (d) human-based, and (e) game-based approaches.

Swarm-based optimization methods were inspired by various natural phenomena and natural behaviors of living organisms in nature. Particle swarm optimization (PSO) is among the oldest and most extensively used algorithms in this category, and it was designed based on natural fish and bird behaviors [9]. Ant colony optimization (ACO) is another swarm-based technique that is focused on simulating ants’ behavior as they travel between nests and food sources, as well as the placement of pheromones in their paths. The presence of more pheromones in a path indicates that the path is closer to the food source [10]. The bat algorithm (BA) is designed by imitating the activity of bats’ sound systems in locating prey, obstacles, and nests [11]. Grey wolf optimization is a nature-based technique that models the hierarchical structure of grey wolves’ social behavior during hunting [12]. Some of the other swarm-based optimization algorithms are green anaconda optimization (GOA) [13], the spotted hyena optimizer (SHO) [14], northern goshawk optimization (NGO) [15], the orca predation algorithm (OPA) [16], the artificial fish-swarm algorithm (AFSA) [17], the reptile search algorithm (RSA) [18], the firefly algorithm (FA) [19], the grasshopper optimization algorithm (GOA) [20], dolphin partner optimization (DPO) [21], the whale optimization algorithm (WOA) [22], the hunting search (HS) [23], moth–flame optimization (MFO) [24], the seagull optimization algorithm (SOA) [25], the subtraction-average-based optimizer (SABO) [26], the remora optimization algorithm (ROA) [27], the marine predators algorithm (MPA) [28], the artificial hummingbird algorithm (AHA) [29], red fox optimization (RFO) [30], the tunicate swarm algorithm (TSA) [31], the pelican optimization algorithm (POA) [32], the cat- and mouse-based optimizer (CMBO) [33], the selecting-some-variables-to-update-based algorithm (SSVUBA) [34], the good, the bad, and the ugly optimizer (GBUO) [35], the group mean-based optimizer (GMBO) [36], and the snake optimizer (SO) [37].

Physics-based optimization algorithms are produced by drawing inspiration from numerous physical occurrences and using a variety of its rules. Simulated annealing (SA) is one of the methods in this group that originates from the process of refrigerating molten metals. In the refrigeration process, a very high-temperature molten metal is gradually cooled [38]. The gravitational search algorithm (GSA) is designed by modeling the force of gravity and Newton’s laws of motion in an artificial system in which masses apply force to each other at different distances and move in this system according to such laws [39]. Some of the other physics-based optimization algorithms are the galaxy-based search algorithm (GbSA) [40], the small world optimization algorithm (SWOA) [41], Henry gas solubility optimization (HGSO) [42], central force optimization (CFO) [43], ray optimization (RO) [44], the flow regime algorithm (FRA) [45], curved space optimization (CSO) [46], the billiards-inspired optimization algorithm (BOA) [47], and nuclear reaction optimization (NRO) [48].

Evolutionary-based optimization algorithms are based on the simulation of biological evolution and the theory of natural selection. This category includes the genetic algorithm (GA), one of the earliest approximation optimizers. The GA was developed by modeling the reproductive process according to Darwin’s theory of evolution using three operators: (a) selection, (b) crossover, and (c) mutation [49]. Some of the other evolutionary-based optimization algorithms are the biogeography-based optimizer (BBO) [50], the memetic algorithm (MA) [51], evolutionary programming (EP) [52], the drawer algorithm (DA) [53], evolution strategy (ES) [54], differential evolution (DE) [55], and genetic programming (GP) [56].

Human-based optimization algorithms are developed based on modeling human behavior. Teaching–learning-based optimization (TLBO) is among the most employed human-based algorithms and models the educational process in the classroom between teachers and students. In the TLBO, the educational process is implemented in two phases: (a) a teaching phase in which the teacher shares knowledge with the students and (b) a learner phase in which the students share knowledge with each other [57]. Some of the other human-based optimization algorithms are the mother optimization algorithm (MOA) [58], the exchange market algorithm (EMA) [59], the group counseling optimizer (GCO) [60], the teamwork optimization algorithm (TOA) [6], dual-population social group optimization (DPSGO) [61], and the election-based optimization algorithm (EBOA) [6].

Game-based optimization algorithms originate from the rules of various groups or individual games. The volleyball premier league (VPL) algorithm is based on modeling the interaction and competition among volleyball teams during a season and the coaching process during a match [62]. Some of the other game-based optimization algorithms are football game-based optimization (FGBO) [63], ring toss game-based optimization (RTGBO) [64], the golf optimization algorithm (GOA) [65], and shell game optimization (SGO) [66].

Some other recently proposed metaheuristic algorithms are monarch butterfly optimization (MBO) [67], the slime mold algorithm (SMA) [68], the moth search algorithm (MSA) [69], the Hunger Games search (HGS) [70], the Runge Kutta method (RUN) [71], the colony predation algorithm (CPA) [72], the weighted mean of vectors (INFO) [73], Harris Hawks optimization (HHO) [74], and the Rime optimization algorithm (RIME) [75].

## 3. One-to-One Based Optimizer

In this section, the proposed OOBO algorithm is described, and its mathematical modeling is presented. OOBO is a population-based metaheuristic algorithm that can provide effective solutions to optimization problems in an iteration-based process using a population search power in the problem-solving space.

### 3.1. Basis of the Algorithm

The basis of OOBO is that, first, several feasible solutions are generated based on the constraints of the problem. Then, in each iteration, the position of these solutions in the search space is updated, employing the algorithm’s main idea. Excessive reliance on specific population members in the update process prevents accurate scanning of the problem’s search space. This can lead to the convergence of the algorithm towards local optimal areas. The main idea in designing the proposed OOBO algorithm, while preventing it from relying too much on specific members of the population, such as best, worst, and mean members, is the effective use of information on all population members in the process of updating the algorithm population. Therefore, in this process of updating, the following items are considered: (a) the non-reliance of population updates on its specific members; (b) the involvement of all members in the updating process; and (c) each population member is employed in a one-to-one correspondence to guide another member in the search space.

### 3.2. Algorithm Initialization

In the OOBO algorithm, each population member is a proposed solution to the given problem as values for the decision variables, depending on its location in the search space. As a result, in OOBO, each population member is mathematically represented by a vector with the same number of elements as the number of decision variables. A population member can be represented using

(5) $${\vec{X}}_{i}=\left[x_{i,1},\;\ldots ,\;x_{i,d},\;\ldots ,\;x_{i,m}\right],\;i=1,\;\ldots ,\;N.$$

To generate the initial population of OOBO, population members are randomly positioned in the search space utilizing

(6) $$x_{i,d}=lb_{d}+rand()\cdot \left(ub_{d}-lb_{d}\right),\;d=1,\;\ldots ,m,$$

where ${\vec{X}}_{i}$ is the ith population member (that is, the proposed solution), $x_{i,d}$ is its dth dimension (that is, the proposed value for the dth variable), rand() is a function generating a random uniform number from the interval $\left[0,\;1\right],$ and *N* is the size of the population.

In OOBO, the algorithm population is represented using a matrix according to

(7) $$\vec{X}=\left[\begin{array}{c} {\vec{X}}_{1}\\ \vdots \\ {\vec{X}}_{i}\\ \vdots \\ {\vec{X}}_{N} \end{array}\right]={\left[\begin{array}{ccccc} x_{1,1} & \cdots & x_{1,d} & \cdots & x_{1,m}\\ \vdots & \ddots & \vdots & ⋰ & \vdots \\ {\;x}_{i,1} & \cdots & x_{i,d} & \cdots & x_{i,m}\\ \vdots & ⋰ & \vdots & \ddots & \vdots \\ x_{N,1} & \cdots & x_{N,d} & \cdots & x_{N,m} \end{array}\right]}_{N\times m}.$$

The optimization problem’s objective function can be assessed based on each population member, which is a proposed solution. Thus, different values for the objective function are acquired in each iteration equal to the number of population members, which can be mathematically described by means of

(8) $$\vec{F}={\left[\begin{array}{c} f_{1}\\ \vdots \\ f_{i}\\ \vdots \\ f_{N} \end{array}\right]}_{N\times 1}={\left[\begin{array}{c} f({\vec{X}}_{1})\\ \vdots \\ f({\vec{X}}_{i})\\ \vdots \\ f({\vec{X}}_{N}) \end{array}\right]}_{N\times 1},$$

where $\vec{F}$ is the objective function vector and $f_{i}$ is the objective function value for the ith proposed solution.

### 3.3. Mathematical Modeling of OOBO

At this stage of mathematical modeling for the OOBO algorithm, the population members’ positions must be updated in the search space. The main difference between metaheuristic algorithms is in how to update the position of population members. One of the things that can be seen in many metaheuristic algorithms is that the population update process is strongly dependent on the best member. This may lead to a decrease in the algorithm’s exploration ability to provide the global search in the problem-solving space and then get stuck in the local optimum. In fact, moving the population towards the best member can cause convergence to inappropriate local solutions, especially in complex optimization problems. Meanwhile, in the design of OOBO, the dependence of the population update process on the best member has been prevented. Hence, by moving the population of the algorithm to different areas in the search space, the exploration power of OOBO can be increased to provide the global search. The main idea of OOBO for this process is that all members of the population should participate in population updating. Therefore, each population member is selected only once and randomly to guide a different member of the population in the search space. We can mathematically describe this idea using an N-tuple with the following properties: (a) each member is randomly selected from the positive integers from 1 to N; (b) there are no duplicate members among its members; and (c) no member has a value equal to its position in this N-tuple.

To model a one-to-one correspondence, the member position number in the population matrix is used. The random process of forming the set $\vec{K}$ as “the set of the positions of guiding members” is modeled by

(9) $$\vec{K}=\left\{\left[k_{1},{\ldots ,k}_{l},\ldots ,k_{N}\right]\in P_{\bar{N}};\;{\forall l\in \bar{N}:k}_{l}\neq l\right\},$$

where $\bar{N}=\left\{1,\;\ldots ,\;N\right\},P_{\bar{N}}$ is the set of all permutations of the set $\bar{N}$, and $k_{l}$ is the lth element of the vector $\vec{K}$.

In OOBO, to guide the ith member ($X_{i}$), a member of the population with position number $k_{i}$ ($X_{k_{i}}$) in the population matrix is selected. Based on the values of the objective function of these two members, if the status of member $X_{k_{i}}$ in the search space is better than that of member $X_{i}$, member $X_{i}$ moves to member ${X_{k}}_{i}$; otherwise, it moves away from member ${X_{k}}_{i}$. Based on the above concepts, the process of calculating the new status of population members in the search space is modeled, employing

(10) $$x_{i,d}^{new}=\left\{\begin{array}{c} x_{i,d}+rand()\cdot (x_{k_{i},d}-I\;x_{i,d}),\;f_{k_{i}}<f_{i};\\ x_{i,d}+rand()\cdot (x_{i,d}-x_{k_{i},d}),\;otherwise, \end{array}\right.$$

(11) I=round(1+rand()),

where $x_{i,d}^{new}$ is the new suggested status of the ith member in the dth dimension, $x_{k_{i},d}$ is the dth dimension of the selected member to guide the ith member, $f_{k_{i}}$ is the objective function value obtained based on $X_{k_{i}}$, and the variable I takes values from the set $\left\{1,2\right\}$.

The updating process of the population members in the proposed algorithm is such that the suggested new status for a member is acceptable if it leads to an improvement in the value of the objective function. Otherwise, the suggested new status is unacceptable, and as a result, the member stays in the previous position. This step of modeling OOBO is formulated as

(12) $$X_{i}=\left\{\begin{array}{c} X_{i}^{new},\;f_{i}^{new}<f_{i};\\ X_{i},\;otherwise, \end{array}\right.$$

where $X_{i}^{new}$ is the new suggested status in the search space for the ith population member and $f_{i}^{new}$ is its value of the objective function.

### 3.4. Repetition Process, Pseudocode, and Flowchart of OOBO

At this stage of OOBO, after updating the positions of all members of the population in the search space, the algorithm completes one iteration and enters the next iteration based on the population members’ new statuses. The procedure of updating population members is repeated using Equations (9)–(12) until the algorithm reaches the stopping rule. OOBO provides the best-found solution as a quasi-optimal after fully implementing the algorithm in the given problem. The implementation steps of OOBO are presented as pseudocode in Algorithm 1. The complete set of codes is available at the following repository: https://www.mathworks.com/matlabcentral/fileexchange/135807-one-to-one-based-optimizer-oobo (accessed on 22 September 2023).
**Algorithm 1.** Pseudocode of OOBO.Start OOBO.1. Input optimization problem information.2. Set *N* and *T.*3. Create an initial population matrix.4. Evaluate the objective function.5. for t ← 1 to T do6.  Update $\vec{K}$ based on Equation (9).7.  for i ← 1 to N do8.    Calculate $X_{i}^{new}$ based on Equations (10) and (11).9.    Compute $f_{i}^{new}$ based on $X_{i}^{new}$.10.    Update $X_{i}$ using Equation (12).11.    end for 12.    Save the best solution found so far.13. end for 14. Output the best quasi-optimal solution.End OOBO.

### 3.5. Computational Complexity of OOBO

Next, the computational complexity of the OOBO algorithm, including the time complexity and space complexity, is studied.

The time complexity of OOBO is affected by the initialization process, the calculation of the objective function, and population updating as follows:

- The algorithm initialization process requires O(Nm) time, where, as mentioned, *N* is the number of population members and *m* the number of decision variables.
- In each iteration, the objective function is calculated for each population member. Therefore, calculating the objective function requires O(NT) time, where *T* is the number of iterations of the algorithm.
- The updating of population members requires an O(NTm) time.

Therefore, O(N(T(1+m)+m)) is the total time complexity of the OOBO algorithm, which can be simplified to O(NTm). Competitor algorithms such as GA, PSO, GSA, GWO, WOA, TSA, and MPA have a time complexity equal to $O\left(NTm\right),$ and TLBO has a time complexity equal to O(N(2T(1+m)+m)). Of course, considering that it is usually expressed as time complexity without constants and slower-growing terms, this expression is simplified to O(NTm). Thus, the proposed OOBO approach has a similar time complexity to the seven competitor algorithms mentioned above. Compared to the TLBO, the OOBO approach has less time complexity and better conditions from this perspective.

The space complexity of the OOBO algorithm is O(Nm), which is considered the maximum amount of space in its initialization process. Similarly, the competitor algorithms also have a space complexity equal to O(Nm). In this respect, there is no difference between OOBO and the competitor algorithms.

## 4. Simulation Studies and Results

In this section, OOBO’s ability to solve optimization problems and provide quasi-optimal solutions is evaluated. For this purpose, OOBO was tested on 52 objective functions, which were categorized into (a) seven unimodal functions of $F_{1}$ to $F_{7}$, (b) six high-dimensional multimodal functions of $F_{8}$ to $F_{13}$, and (c) ten fixed-dimensional multimodal test functions of $F_{14}$ to $F_{23}$, as well as twenty-nine functions from the CEC 2017 test suite (C17-F1, C17-F3 to C17-F30). Detailed information and a complete description of the benchmark functions for functions $F_{1}$ to $F_{23}$ are provided in [76], and for the CEC 2017 test suite, they are provided in [77]. In addition, the performance of OOBO was evaluated in four real-world optimization problems.

### 4.1. Intuitive Analysis in Two-Dimensional Search Space

**Figure 1:**
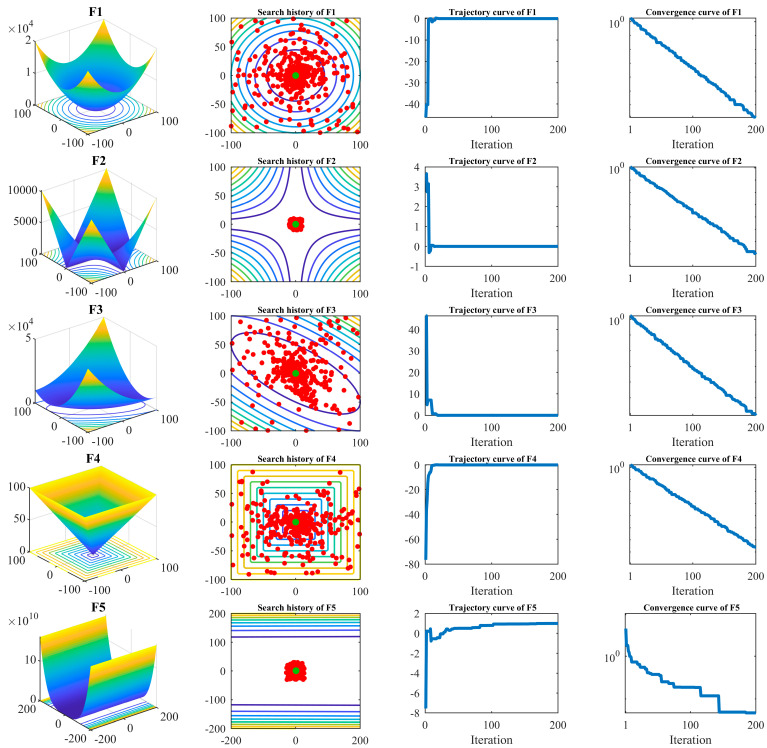

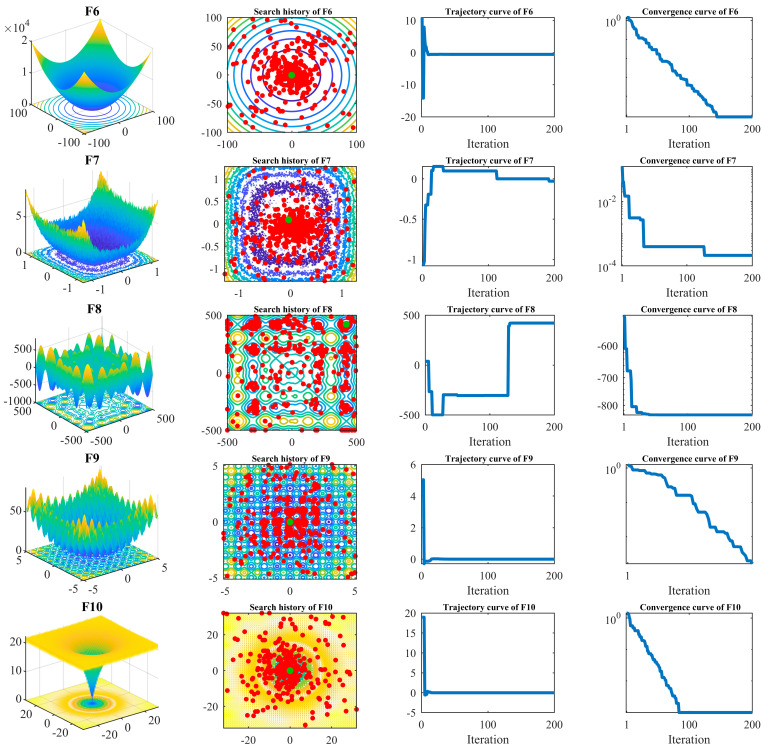
Search history curves, trajectory curves, and convergence curves for optimization of different objective functions using OOBO.

Next, to visually observe the optimization process of the OOBO approach, the OOBO function was implemented in ten objective functions, $F_{1}$ to $F_{10}$, in two dimensions. In this experiment, the number of OOBO population members was considered equal to five. To show the mechanism of the OOBO algorithm in solving the problems related to $F_{1}$ to $F_{10}$, convergence curves, search history curves, and trajectory curves are presented in Figure 1. The horizontal axis in convergence curves and trajectory curves represents the number of iterations of the algorithm. These curves display OOBO’s behavior in scanning the problem-search space, solution-finding, the convergence process, and how it achieves better solutions based on update processes after each iteration, as well as decreasing the objective function values. What was concluded from the analysis of this experiment is that the OOBO approach, by improving the initial candidate solutions during the progress of the algorithm iterations, can converge towards the optimal solution, providing acceptable quasi-optimal solutions for the given problem.

### 4.2. Experimental Setup

To further analyze the quality of OOBO, the results obtained from this algorithm were compared with eight well-known optimization algorithms: PSO, TLO, GWO, WOA, MPA, TSA, GSA, and GA. The reasons for choosing these competitor algorithms were as follows: GA and PSO are among the most famous and widely used optimization algorithms that have been employed in many applications; GSA, TLBO, and GWO are highly cited algorithms, which shows that they have always been trusted and used by researchers. Additionally, WOA, MPA, and TSA are methods that have been published recently, and because of their acceptable performance, they have been favored by many researchers in this short period of publication. Therefore, in total, eight competitor algorithms in this study were selected, based on the following three criteria:

(i) The most widely used algorithms: GA and PSO.
(ii) Highly cited algorithms: GSA, TLBO, and GWO.
(iii) Recently published and widely used algorithms: WOA, MPA, and TSA.

The values used for the control parameters of these competitors are specified in Table 1. To provide a fair comparison, standard versions of metaheuristic algorithms are used. Experiments were implemented on the software MATLAB R2022a utilizing a 64-bit Core i7 processor with 3.20 GHz and 16 GB main memory. 

### 4.3. Performance Comparison

The ability of OOBO was compared with eight competitor algorithms applied to different objective functions of unimodal and multimodal types. Five indicators (mean, best, worst, standard deviation, and median) of the best-found solutions were used to report the performance results of the algorithms. To optimize each of the objective functions, OOBO was implemented in 20 independent runs, each of which contained 1000 iterations. Convergence curves for each benchmark function were drawn based on the average performance of metaheuristic algorithms in 20 independent runs. Random optimization algorithms are stochastic-based approaches that can provide a solution to the problem in an iterative process. An essential point in implementing optimization algorithms is determining the stopping rule for the algorithm iterations. There are various stopping rules (criteria) for optimization algorithms, including the total number of iterations, the total number of function evaluations, no change in the value of the objective function after a certain number of iterations, and determining an error level between the values of the objective function in several consecutive repetitions. Among them, the total number of iterations has been the focus of researchers, who employ this criterion for the stopping rule. Hence, the present investigation considered the total number of iterations (*T*) as a stopping rule for optimization algorithms in solving the functions *F*_1_ to *F*_23_ and function evaluations (FEs) in solving the CEC 2017 test suite.

Seven unimodal structures were included in the first group of objective functions analyzed to assess the competence of OOBO. Table 2 reports the implementation results of OOBO and eight competitors. What is clear from the analysis of the simulation results is that OOBO is the first best optimizer for the functions $F_{1}$, $F_{2}$, $F_{3}$, $F_{4}$, $F_{5}$, $F_{6}$, and $F_{7}$ compared to the competitor algorithms. The comparison of the simulation results demonstrates that the proposed OOBO has a great capacity to solve unimodal problems and is far more competitive than the other eight algorithms.

The second set of objective functions chosen to assess the efficacy of optimization algorithms consisted of six high-dimensional multimodal objective functions, $F_{8}$ to $F_{13}$. Table 3 presents the outcomes of optimizing these objective functions utilizing the proposed OOBO and eight competitor techniques. Based on the simulation results, OOBO provides the optimal solution for $F_{9}$ and $F_{11}$ and is also the first-best optimizer for $F_{8}$, $F_{10}$, $F_{12}$, and $F_{13}$. Similarly, it was determined that OOBO has a more efficient ability to provide suitable solutions for $F_{8}$ to $F_{13}$ in relation to the competitor algorithms.

Ten fixed-dimensional multimodal functions were considered as the third group of objective functions to test the performance of the optimization techniques. Table 4 provides the outcomes of implementing the proposed OOBO and eight competitor algorithms on $F_{14}$ to $F_{23}$. The simulation results reveal that OOBO outperforms the competitor algorithms for $F_{14}$, $F_{15}$, $F_{20}$, $F_{21}$, $F_{22}$, and $F_{23}$. In optimizing the functions $F_{16}$, $F_{17}$, $F_{18}$, and $F_{19}$, although from the “mean” perspective, the performance of several algorithms is the same, OOBO has better “standard deviation,” providing adequate solutions. The simulation results demonstrate that OOBO is more efficient than the competitor algorithms at solving this sort of objective functions.

**Figure 2:**
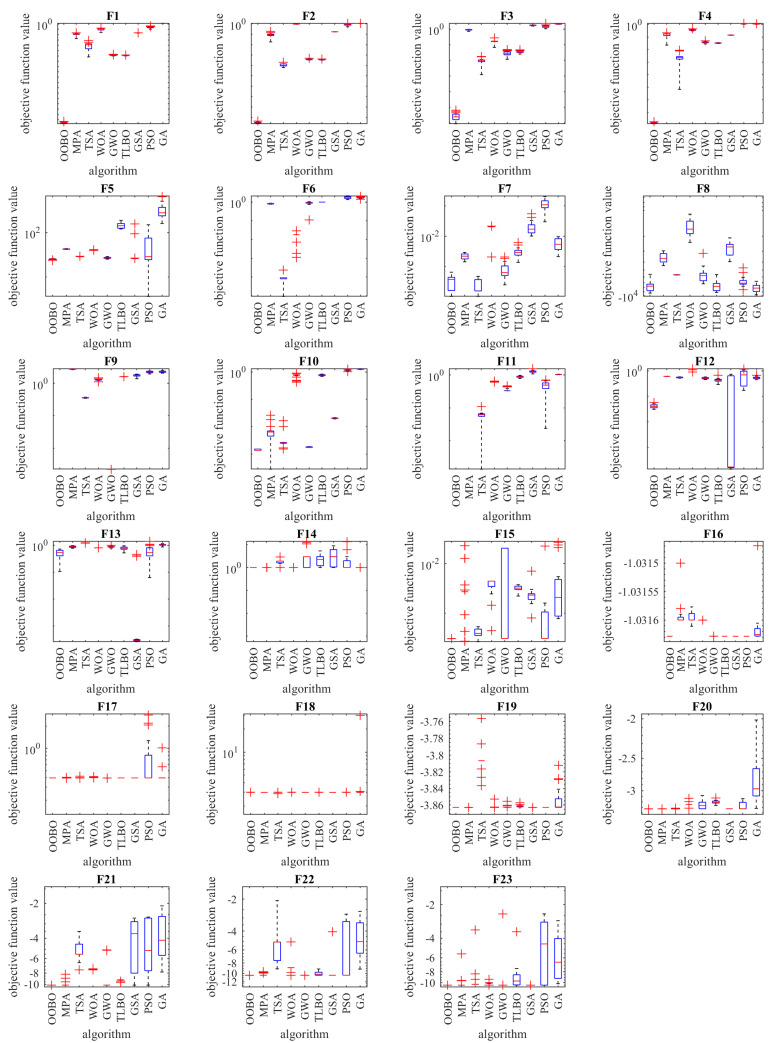
Boxplots of the performance of OOBO and competitor algorithms based on $F_{1}$ to $F_{23}$.

**Figure 3:**
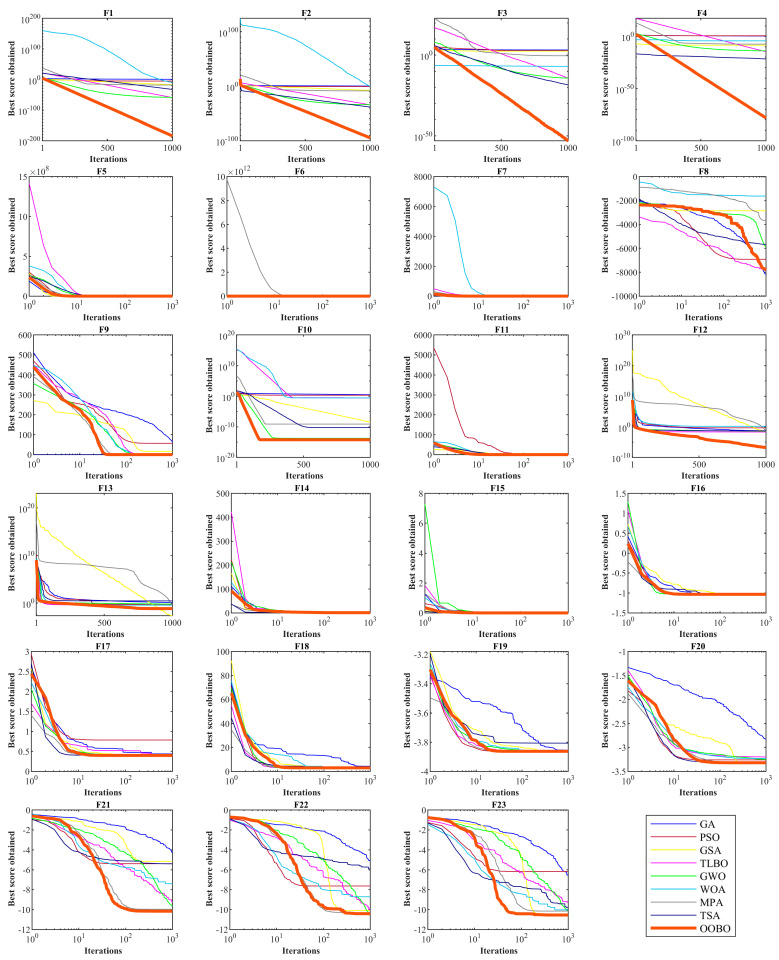
Convergence curves of OOBO and competitor algorithms in optimizing $F_{1}$ to $F_{23}$.

Figure 2 depicts a boxplot of the performance of optimization algorithms in solving objective functions $F_{1}$ to $F_{23}$. In addition, the convergence curves of the OOBO approach and all competitor algorithms for benchmark functions $F_{1}$ to $F_{23}$ are presented in Figure 3. The best score in convergence curves refers to the best value obtained for the objective function up to each iteration. This index is updated in each iteration based on the comparison with its value in the previous iteration. The analysis of the convergence curves indicates that, when solving unimodal problems with objectives functions $F_{1}$ to $F_{7}$, the proposed OOBO converges on much better solutions than its eight competitor algorithms, and it has superior performance. When solving high-dimensional multi-model problems based on $F_{8}$ to $F_{13}$, OOBO has a greater convergence strength than its eight competitor algorithms. When solving high-dimensional multi-model problems using $F_{14}$ to $F_{23}$, the proposed OOBO approach has a faster convergence speed and greater convergence strength than eight competitor algorithms. 

### 4.4. Sensitivity Analysis

**Figure 4:**
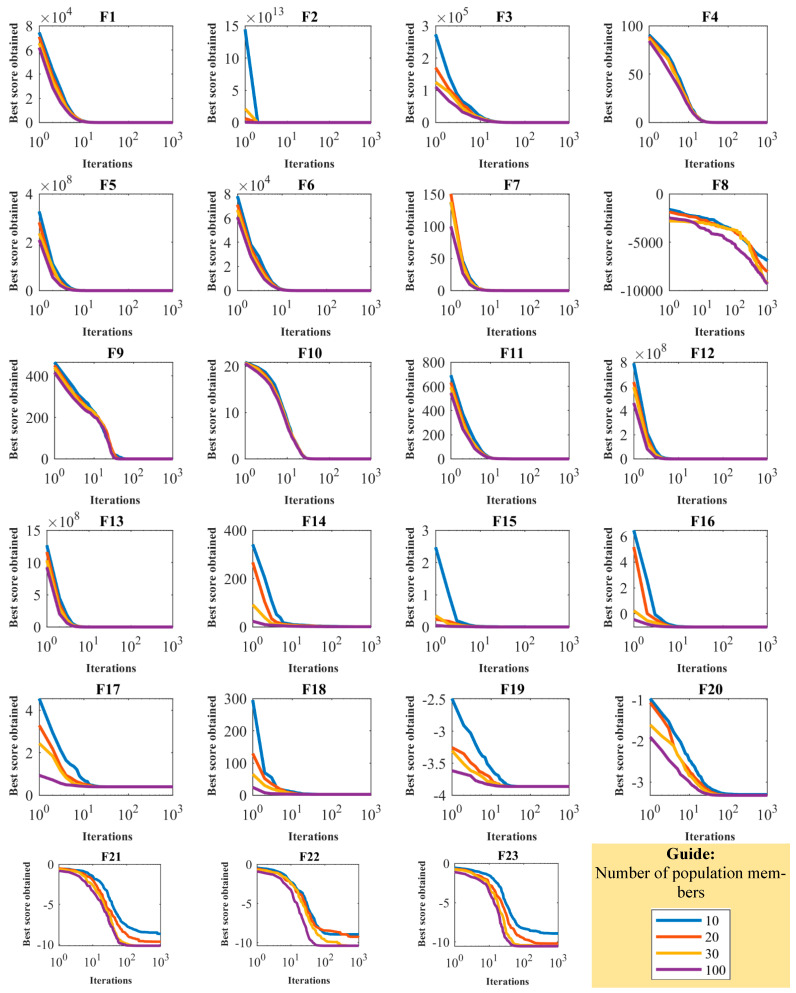
Convergence curves of sensitivity analysis of OOBO in relation to N.

**Figure 5:**
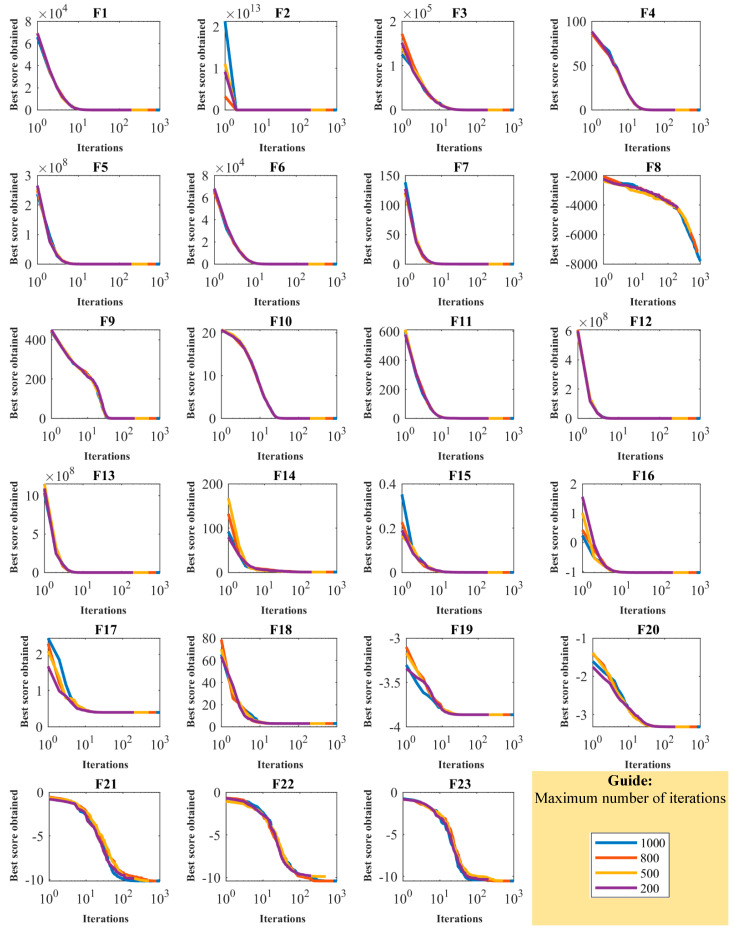
Convergence curves of sensitivity analysis of OOBO in relation to T.

The proposed OOBO employs two parameters, the number of population members (N) and the maximum number of iterations (T) in the implementation process, to solve optimization problems. In this regard, the analysis of OOBO’s sensitivity to these two parameters was assessed next. OOBO was implemented in independent runs for different values of N = 10, 20, 30, and 100 on $F_{1}$ to $F_{23}$ to investigate the sensitivity of the proposed method to the number of population members’ parameter. The simulation results of this part of the study are reported in Table 5, whereas the behavior of the convergence curves under the impact of changes in population size is displayed in Figure 4. The simulation results show that the values of all objective functions decline as the population size increases. To investigate the proposed algorithm’s sensitivity in relation to T, OOBO is employed in independent runs with different values of this parameter equal to *T* = 200, 500, 800, and 1000 for optimizing the functions $F_{1}$ to $F_{23}$. Table 6 and Figure 5 show the results of the sensitivity analysis of OOBO regarding T. The inference from the OOBO sensitivity analysis with the parameter T is that this algorithm can converge on better optimal solutions when employed in a larger number of iterations.

### 4.5. Scalability Analysis

**Figure 6:**
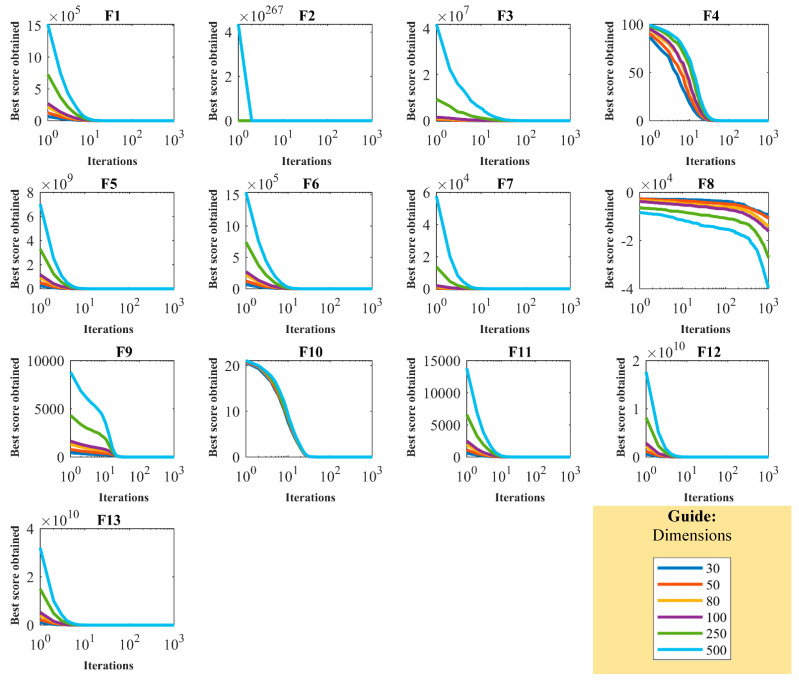
Conversance curves of scalability study results of OOBO.

Next, a scalability study is presented to analyze the performance of OOBO in optimizing objective functions under the influence of changes in the problem dimensions. For this purpose, OOBO was employed in different dimensions (30, 50, 80, 100, 250, and 500) in optimizing $F_{1}$ to $F_{13}$. The OOBO convergence curves in solving objective functions for the various mentioned dimensions are presented in Figure 6. The simulation results obtained from the scalability study are reported in Table 7. From the analysis of the results in this table, we can deduce that the efficiency of OOBO is not degraded too much when the dimensions of the given problem increase. OOBO’s optimal performance under the influence of changes in the problem dimensions is due to OOBO’s ability to achieve the proper balance between exploration and exploitation.

### 4.6. Evaluation of the CEC 2017 Test Suite 

**Figure 7:**
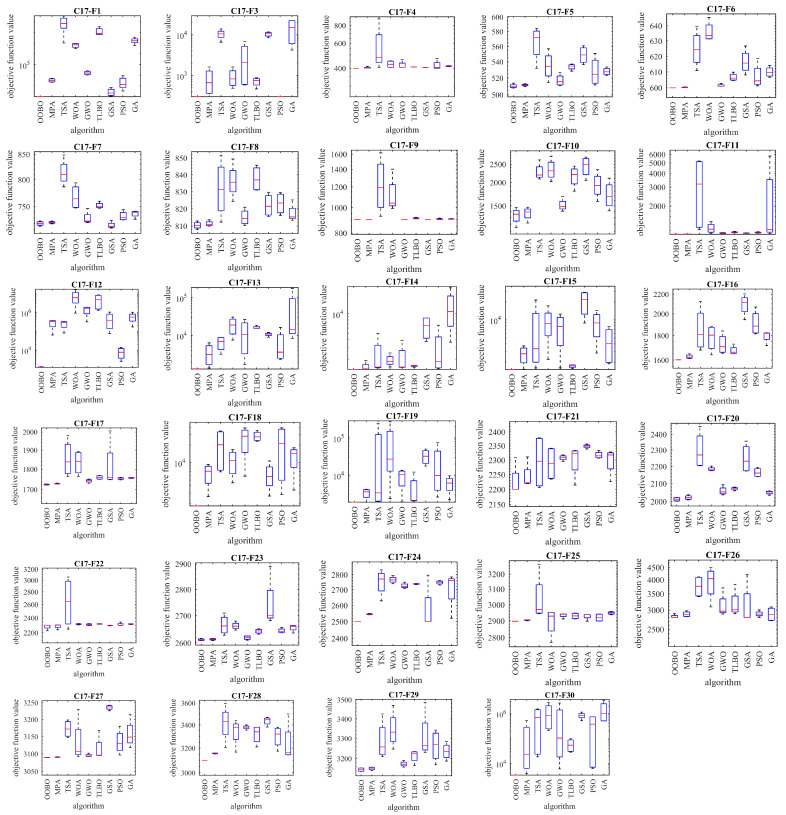
Boxplot diagram of OOBO and competitor algorithms using the CEC 2017 test suite.

Next, the performance of the proposed OOBO approach in handling the CEC 2017 test suite was evaluated. The CEC 2017 test suite has thirty benchmark functions consisting of three unimodal functions, C17-F1 to C17-F3, seven multimodal functions, C17-F4 to C17-F10, ten hybrid functions, C17-F11 to C17-F20, and ten composition functions, C17-F21 to C17-F30. The function C17-F2 was removed from this test suite due to unstable behavior (as in similar papers). The unstable behavior of C17-F2 means that, especially for higher dimensions, it shows significant performance variations for the same algorithm implemented in Matlab. Complete information on the CEC 2017 test suite is provided in [77]. The implementation results of OOBO and competitor algorithms with the CEC 2017 test suite are reported in Table 8. The boxplot diagrams obtained from the performance of the metaheuristic algorithms in handling the CEC 2017 test suite are drawn in Figure 7.

Based on the simulation results, OOBO is the first-best optimizer for the functions C17-F1, C17-F4 to C17-F6, C17-F8, C17-F10 to C17-24, and C17-F26 to C17-F30. The analysis of the simulation results shows that the proposed OOBO approach has been able to provide superior performance in solving the CEC 2017 test suite by providing better results in most of the benchmark functions compared to competitor algorithms.

### 4.7. Statistical Analysis

Next, the Wilcoxon rank sum test [78] was utilized to evaluate the performance of optimization algorithms in addition to statistical analysis of the average and standard deviation. The Wilcoxon rank sum test is used to determine whether there is a statistically significant difference between two sets of data. A *p*-value in this test reveals whether the difference between OOBO and each of the competitor algorithms is statistically significant. Table 9 reports the results of our statistical analysis. Based on the analysis of the simulation results, the proposed OOBO has a *p*-value less than 0.05 in each of the three types of objective functions compared to each of the competitor algorithms. This result indicates that OOBO is significantly different in statistical terms from the eight compared algorithms.

## 5. OOBO for Real-World Applications

In this section, the proposed OOBO and eight competitor algorithms are applied to four science/engineering designs to evaluate their capacity to resolve real-world problems. These design problems are pressure vessel, speed reducer, welded beam, and tension/compression spring.

### 5.1. Pressure Vessel Design Problem

**Figure 8:**
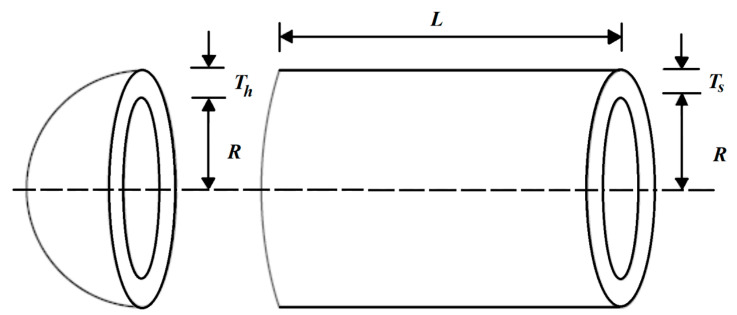
Schematic of the pressure vessel design.

The mathematical model of the pressure vessel design problem was adapted from [79]. The main goal of this problem is to minimize the design cost. A schematic view of the pressure vessel design problem is shown in Figure 8. 

To formulate the model, consider that $X=\left[x_{1},\;x_{2},\;x_{3},\;x_{4}\right]=\left[T_{s},\;T_{h},\;R,\;L\right]$, and then the mathematical program is given by

Minimize:

$$f\left(x\right)=0.6224x_{1}x_{3}x_{4}+1.778x_{2}x_{3}^{2}+3.1661x_{1}^{2}x_{4}+19.84x_{1}^{2}x_{3}$$

Subject to:

$$g_{1}\left(x\right)=-x_{1}+0.0193x_{3}\;\leq \;0,\;$$

$$g_{2}\left(x\right)=-x_{2}+0.00954x_{3}\leq \;0,$$

$$g_{3}\left(x\right)=-\pi x_{3}^{2}x_{4}-\frac{4}{3}\pi x_{3}^{3}+1296000\leq \;0,\;$$

$$g_{4}\left(x\right)=x_{4}-240\;\leq \;0,$$

with $0\leq x_{1},\;x_{2}\leq 100\;{and\;10\leq x}_{3},\;x_{4}\leq 200$.

**Figure 9:**
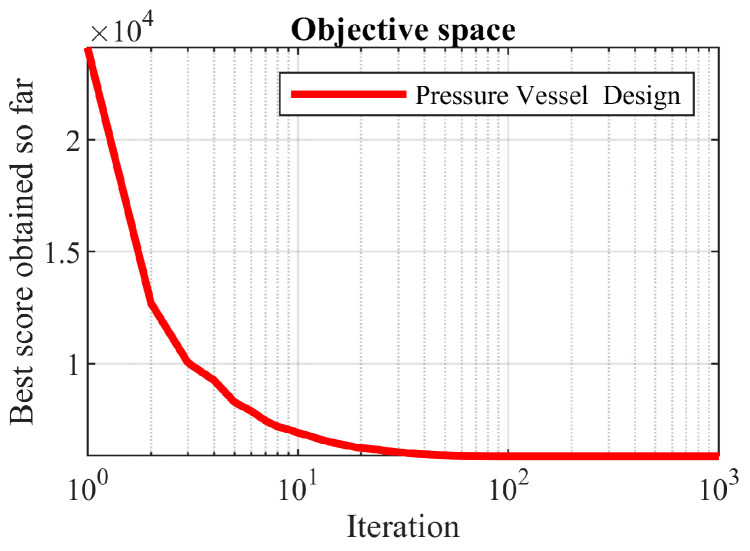
OOBO’s performance convergence curve on the pressure vessel design.

The obtained solutions using OOBO and eight competitor algorithms are presented in Table 10. Based on the results of this table, OOBO presented the optimal solution at (0.7781, 0.3832, 40.3150, 200), and the value of the objective function was equal to 5870.8460. An analysis of the results of this table showed that the proposed OOBO has good performance in solving the problem at a low cost. The statistical results of the performance of the optimization algorithms when solving this problem are presented in Table 11. These results indicated that OOBO provides better values for the best, mean, and median indices than the other eight compared algorithms. The convergence curve of the proposed OOBO is presented in Figure 9 while achieving the optimal solution.

### 5.2. Speed Reducer Design Problem

**Figure 10:**
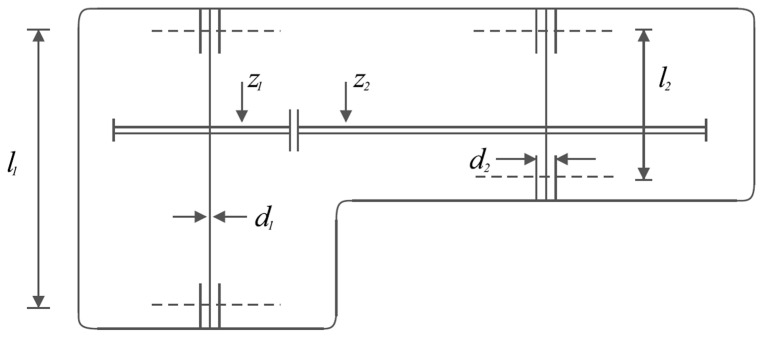
Schematic of the speed reducer design.

The mathematical model of the speed reducer design problem was first formulated in [80], but we used an adapted formulation from [81]. The main goal of this problem is to minimize the weight of the speed reducer. A schematic view of the speed reducer design problem is shown in Figure 10. 

To formulate the model, consider that $X=\left[x_{1,}x_{2},x_{3},x_{4},x_{5}{,x}_{6},x_{7}\right]=\left[b,m,p,l_{1},l_{2},d_{1},d_{2}\right],$ and then the mathematical program is given by

Minimize:

$$f\left(x\right)=0.7854x_{1}x_{2}^{2}\left(3.3333x_{3}^{2}+14.9334x_{3}-43.0934\right)-1.508x_{1}\left(x_{6}^{2}+x_{7}^{2}\right)+7.4777\left(x_{6}^{3}+x_{7}^{3}\right)+0.7854(x_{4}x_{6}^{2}+x_{5}x_{7}^{2})$$

Subject to:

$$g_{1}\left(x\right)=\frac{27}{x_{1}x_{2}^{2}x_{3}}-1\;\leq \;0,\;g_{2}\left(x\right)=\frac{397.5}{x_{1}x_{2}^{2}x_{3}}-1\leq \;0,$$

$$g_{3}\left(x\right)=\frac{1.93x_{4}^{3}}{x_{2}x_{3}x_{6}^{4}}-1\leq \;0,\;g_{4}\left(x\right)=\frac{1.93x_{5}^{3}}{x_{2}x_{3}x_{7}^{4}}-1\;\leq \;0,$$

$$g_{5}\left(x\right)=\frac{1}{110x_{6}^{3}}\sqrt{{\left(\frac{745x_{4}}{x_{2}x_{3}}\right)}^{2}+16.9\cdot 10^{6}}-1\leq \;0,$$

$$g_{6}(x)=\frac{1}{85x_{7}^{3}}\sqrt{{\left(\frac{745x_{5}}{x_{2}x_{3}}\right)}^{2}+157.5\cdot 10^{6}}-1\;\leq \;0,$$

$$g_{7}\left(x\right)=\frac{x_{2}x_{3}}{40}-1\;\leq \;0,\;g_{8}\left(x\right)=\frac{{5x}_{2}}{x_{1}}-1\;\leq \;0,$$

$$g_{9}\left(x\right)=\frac{x_{1}}{12x_{2}}-1\;\leq \;0,\;$$

$$g_{10}\left(x\right)=\frac{{1.5x}_{6}+1.9}{x_{4}}-1\;\leq \;0,$$

$$\;g_{11}\left(x\right)=\frac{{1.1x}_{7}+1.9}{x_{5}}-1\;\leq \;0,$$

with

$$2.6\leq x_{1}\leq 3.6,\;0.7\leq x_{2}\leq 0.8,\;17\leq x_{3}\leq 28,\;7.3\leq x_{4}\leq 8.3,\;7.8\leq x_{5}\leq 8.3,\;2.9\leq x_{6}\leq 3.9$$

and

$$5\leq x_{7}\leq 5.5.$$

**Figure 11:**
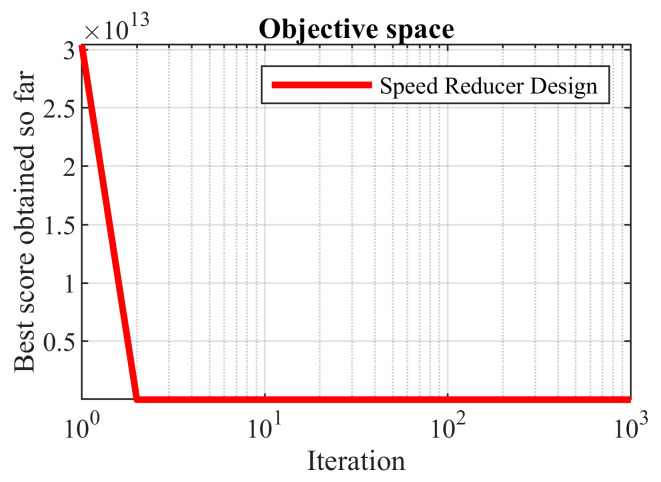
OOBO’s performance convergence curve on the speed reducer design.

The results of the implementation of the proposed OOBO and eight compared algorithms on this problem are presented in Table 12. OOBO presented the optimal solution at (3.5012, 0.7, 17, 7.3, 7.8, 3.33412, 5.26531) with an objective function value of 2989.8520. Table 13 presents the statistical results obtained from the proposed OOBO and eight competitor algorithms. Based on the simulation results, OOBO has superior performance over the eight algorithms when solving the speed reducer design problem. The convergence curve of the proposed OOBO is presented in Figure 11.

### 5.3. Welded Beam Design

**Figure 12:**
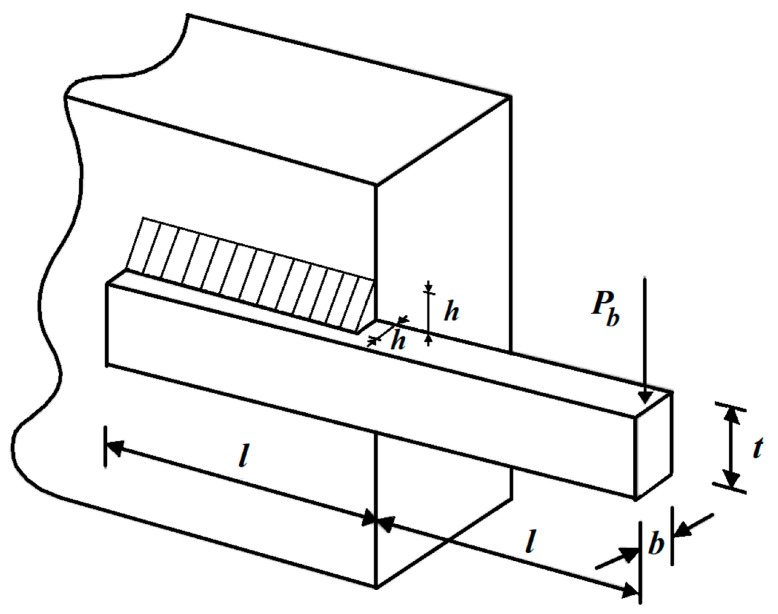
Schematic of the welded beam design.

The mathematical model of a welded beam design was adapted from [22]. The main goal for solving this design problem is to minimize the fabrication cost of the welded beam. A schematic view of the welded beam design problem is shown in Figure 12. 

To formulate the model, consider that $X=\left[x_{1},\;x_{2},\;x_{3},\;x_{4}\right]=\left[h,\;l,\;t,\;b\right]$, and then the mathematical program is given by

Minimize:

$$f(x)=1.10471x_{1}^{2}x_{2}+0.04811x_{3}x_{4}\;(14.0+x_{2})$$

Subject to:

$$g_{1}\left(x\right)=\tau \left(x\right)-13600\;\leq \;0,\;$$

$$g_{2}\left(x\right)=\sigma \left(x\right)-30000\;\leq \;0,$$

$$g_{3}\left(x\right)=x_{1}-x_{4}\leq \;0,\;$$

$$g_{4}(x)=0.10471x_{1}^{2}+0.04811x_{3}x_{4}\;(14+x_{2})-5.0\;\leq \;0,$$

$$g_{5}\left(x\right)=0.125-x_{1}\leq \;0,\;$$

$$g_{6}\left(x\right)=\delta \;\left(x\right)-0.25\;\leq \;0,$$

$$g_{7}\left(x\right)=6000-p_{c}\;\left(x\right)\leq \;0,$$

where

$$\tau \left(x\right)=\sqrt{{\left(\tau^{\prime }\right)}^{2}+\left(2\tau \tau^{\prime }\right)\frac{x_{2}}{2R}+{\left(\tau ”\right)}^{2}\;},\;\tau^{\prime }=\frac{6000}{\sqrt{2}x_{1}x_{2}},\;\tau ”=\frac{MR}{J},$$

$$M=6000\left(14+\frac{x_{2}}{2}\right),\;R=\sqrt{\frac{x_{2}^{2}}{4}+{\left(\frac{x_{1}+x_{3}}{2}\right)}^{2}},$$

$$J=2\sqrt{2}x_{1}x_{2}\left(\frac{x_{2}^{2}}{12}+{\left(\frac{x_{1}+x_{3}}{2}\right)}^{2}\right)\;,\;\sigma \left(x\right)=\frac{504000}{x_{4}x_{3}^{2}}\;,\delta \;\left(x\right)=\frac{65856000}{\left(30\cdot 10^{6}\right)x_{4}x_{3}^{3}}\;,$$

$$\;p_{c}\;\left(x\right)=\frac{4.013\left(30\cdot 10^{6}\right)x_{3}x_{4}^{3}}{1176}\left(1-\frac{x_{3}}{28}\sqrt{\frac{30\cdot 10^{6}}{4(12\cdot 10^{6})}}\right)\;,$$

with $0.1\leq x_{1},x_{4}\leq 2and0.1\leq x_{2},x_{3}\leq 10$.

**Figure 13:**
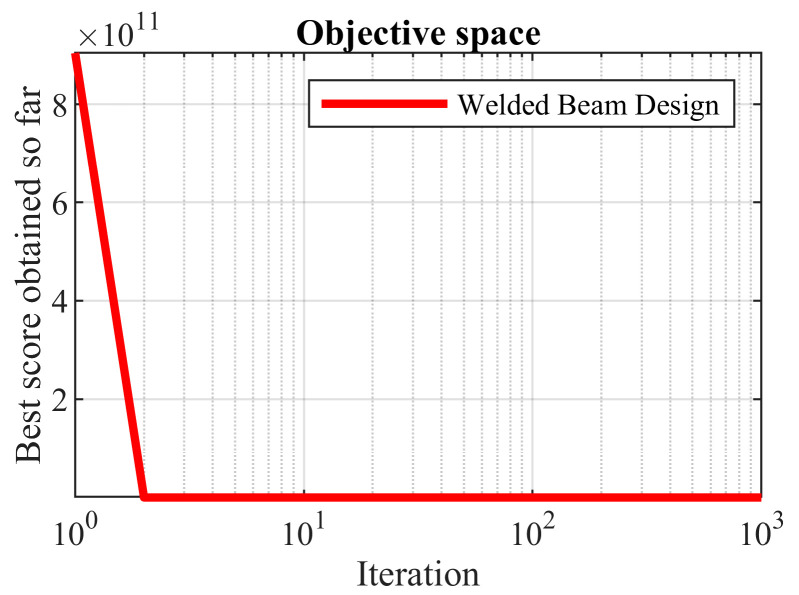
OOBO’s performance convergence curve on the welded beam design.

The results of the implementation of the proposed OOBO and the compared algorithms on this problem are presented in Table 14. The simulation results show that the proposed algorithm presented the optimal solution at (0.20328, 3.47115, 9.03500, 0.20116) with an objective function value of 1.72099. An analysis of the statistical results of the implemented algorithms is presented in Table 15. Based on this analysis, note that the proposed OOBO is superior to the compared algorithms in providing the best, mean, and median indices. The convergence curve of OOBO to solve the welded beam design problem is shown in Figure 13.

### 5.4. Tension/Compression Spring Design

**Figure 14:**
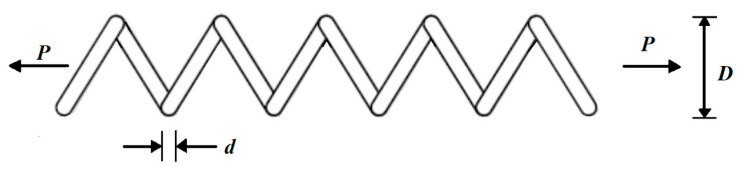
Schematic of the tension/compression spring design.

The mathematical model of this problem was adapted from [22]. The main goal of this design problem is to minimize the tension/compression of the spring weight. A schematic view of the tension/compression spring design problem is shown in Figure 14. 

To formulate the model, consider that $X=\left[x_{1},\;x_{2},\;x_{3}\;\right]=\left[d,\;D,\;P\right]$, and then the mathematical program is given by

Minimize:

$$f\left(x\right)=\left(x_{3}+2\right)x_{2}x_{1}^{2}$$

Subject to:

$$g_{1}\left(x\right)=1-\frac{x_{2}^{3}x_{3}}{71785x_{1}^{4}}\;\leq \;0,\;$$

$$g_{2}\left(x\right)=\frac{4x_{2}^{2}-x_{1}x_{2}}{12566(x_{2}x_{1}^{3}-x_{1}^{4})}+\frac{1}{5108x_{1}^{2}}-1\leq \;0,$$

$$g_{3}\left(x\right)=1-\frac{140.45x_{1}}{x_{2}^{2}x_{3}}\leq \;0,\;g_{4}\left(x\right)=\frac{x_{1}+x_{2}}{1.5}-1\;\leq \;0,$$

with $0.05\leq x_{1}\leq 2,{0.25\leq x}_{2}\leq 1.3and2\leq x_{3}\leq 15$.

**Figure 15:**
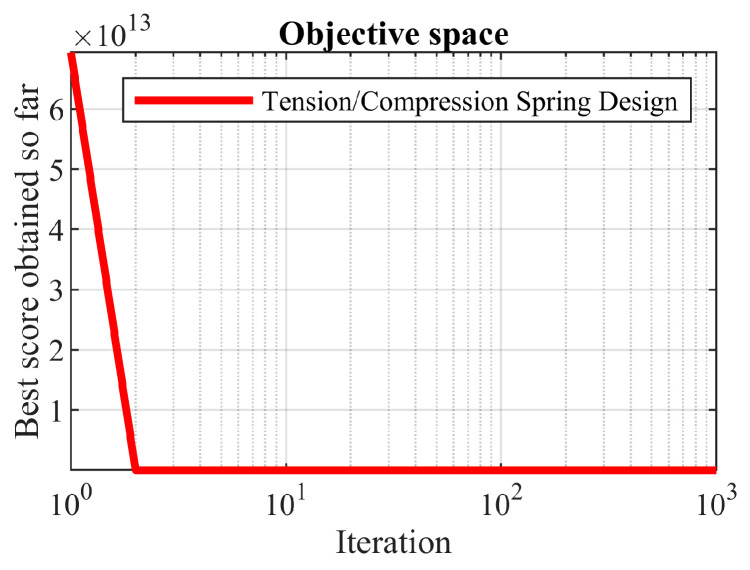
OOBO’s performance convergence curve on the tension/compression spring.

The performance of all optimization algorithms in achieving the objective values and design variables values is presented in Table 16. The optimization results show that the proposed OOBO provided the optimal solution at (0.05107, 0.34288, 12.08809) with an objective function value of 0.01266. A comparison of the results showed that OOBO has superior performance in solving this problem compared to those of the other eight algorithms. A comparison of the statistical results of the performance of the proposed OOBO against the eight competitor algorithms is provided in Table 17. The analysis of this table reveals that OOBO offers a more competitive performance in providing the best, mean, and median indices. The convergence curve of the proposed OOBO in achieving the obtained optimal solution is shown in Figure 15.

## 6. Conclusions and Future Works

A new optimization technique called one-to-one-based optimizer (OOBO) was proposed in this study. The main idea in designing OOBO was the participation of all population members in the algorithm’s updating process based on a one-to-one correspondence between the two sets of members of the population and a set of selected members as guides. Thus, each population member was selected precisely once as a guide to another member and then used to update the position of that population member. The performance of the proposed OOBO in solving optimization problems was tested on 52 objective functions belonging to unimodal, high-dimensional, and fixed-dimensional multimodal types, as well as the CEC 2017 test suite. The findings indicated OOBO’s strong ability in exploitation based on the results of unimodal functions, OOBO’s strong ability in exploration based on the results of high-dimensional multimodal functions, and OOBO’s acceptability in balancing exploitation and exploration based on the results of fixed-dimensional multimodal, hybrid, and composition functions. 

In addition, the performance of the proposed approach in solving optimization problems was compared with eight well-known algorithms. Simulation results reported that the proposed algorithm provided quasi-optimal solutions with better convergence than the compared algorithms. Furthermore, the power of the proposed approach to provide suitable solutions for real-world applications was tested by applying it to four science/engineering design problems. It is clear from the optimization results of this experiment that the proposed OOBO is applicable to solving real-world optimization problems. In response to the main research question about introducing a new optimization algorithm, the simulation findings showed that the proposed OOBO approach performed better in most of the benchmark functions than its competing algorithms. The successful and acceptable performance of OOBO justifies the introduction and design of the proposed approach.

Against advantages such as a strong ability to balance exploration and exploitation and effectiveness in handling real-world applications, the proposed OOBO approach has limitations and disadvantages. The first limitation for all optimization algorithms is that, based on the NFL theorem, there is always a possibility that newer algorithms will be designed that perform better than OOBO. A second limitation of OOBO is that there is no guarantee of achieving global optimization using it due to the nature of random search. Another limitation of OOBO is that, although it has provided successful performance in the optimization problems under study in this paper, there is no guarantee that it will provide similar performance in other optimization applications. Therefore, it is never and in no way claimed that OOBO is the best optimizer for all optimization applications.

The authors of this paper provide several study proposals for the present research. In this regard, we can mention the multi-objective version’s design and the binary version of the proposed OOBO algorithm. Moreover, the usage of OOBO for NP-hard/NP-complete problems, different applications, and optimization problems in science, engineering, data mining, data clustering, sensor placement, big data, medical, and feature selection is additional research potential for further studies based on this paper. 

## Figures and Tables

**Table 1:** Control parameters values.

Algorithm	Parameter	Value
MPA		
	Constant number	P=0.5
	Random vector	*R* is a vector of uniform random numbers from the interval $\left[0,\;1\right].$
	Fish aggregating devices (FADs)	FADs=0.2
	Binary vector	U=0 or 1
TSA		
	$P_{min}$ and $P_{max}$	1 and 4
	$c_{1},c_{2},\;c_{3}$	random numbers lying in the interval $\left[0,\;1\right].$
WOA		
	Convergence parameter (*a*)	*a*: Linear reduction from 2 to 0.
	*r* is a random vector whose components are from the interval $\left[0,\;1\right].$	
	*l* is a random number in $\left[-1,\;1\right].$	
GWO		
	Convergence parameter (*a*)	*a*: Linear reduction from 2 to 0.
TLBO		
	$T_{F}$: teaching factor	$T_{F}=round\;\left[\left(1+rand()\right)\right]$
	random number	*rand* is a random real number from the interval $\left[0,\;1\right]$.
GSA		
	Alpha, *G_0_*, *R_norm_*, *R_power_*	20, 100, 2, 1
PSO		
	Topology	Fully connected
	Cognitive and social constant	$\left(C_{1},\;C_{2})=(2,\;2\right)$
	Inertia weight	Linear reduction from 0.9 to 0.1
	Velocity limit	10% of dimension range
GA		
	Type	Real coded
	Selection	Roulette wheel (proportionate)
	Crossover	Whole arithmetic (probability = 0.8, $\alpha \in \left[-0.5,\;1.5\right]$)
	Mutation	Gaussian (probability = 0.05)

**Table 2:** Optimization results for the indicated algorithm and unimodal functions.

		GA	PSO	GSA	TLBO	GWO	WOA	TSA	MPA	OOBO
$F_{1}$	Mean	13.24055	1.77 × 10^−5^	2.03 × 10^−17^	1.34 × 10^−59^	1.09 × 10^−58^	1.59 × 10^−9^	8.21 × 10^−33^	1.7 × 1010^−18^	3.9 × 10^−185^
	Best	5.593489	2 × 10^−10^	8.2 × 10^−18^	9.36 × 10^−61^	7.73 × 10^−61^	1.09 × 10^−16^	1.14 × 10^−62^	3.41 × 10^−28^	2.3 × 10^−188^
	Worst	27.9284	0.0002	3.87 × 10^−17^	7.65 × 10^−59^	1.841 × 10^−57^	1.085 × 10^−8^	8.7 × 10^−32^	3.04 × 10^−17^	3.10 × 10^−184^
	Std	5.727367	5.86 × 10^−5^	7.1 × 10^−18^	2.05 × 10^−59^	4.09 × 10^−58^	3.22 × 10^−9^	2.53 × 10^−32^	6.76 × 10^−18^	1.27 × 10^−568^
	Median	11.04546	9.92 × 10^−7^	1.78 × 10^−17^	4.69 × 10^−60^	1.08 × 10^−59^	1.09 × 10^−9^	3.89 × 10^−38^	1.27 × 10^−19^	4.4 × 10^−186^
	Rank	9	8	6	4	3	7	2	5	1
$F_{2}$	Mean	2.47941	0.341137	2.37 × 10^−8^	5.55 × 10^−35^	1.3 × 10^−34^	0.53813	5.02 × 10^−39^	2.78 × 10^−9^	1.94 × 10^−95^
	Best	1.591137	0.001741	1.59 × 10^−8^	1.32 × 10^−35^	1.55 × 10^−35^	0.461308	8.26 × 10^−43^	4.25 × 10^−18^	5.82 × 10^−97^
	Worst	4.192926	2.998757	3.13 × 10^−8^	2.07 × 10^−34^	8.61 × 10^−34^	0.612587	7.8 × 10^−38^	4.85 × 10^−8^	1.93 × 10^−94^
	Std	0.642854	0.669594	3.96 × 10^−9^	4.71 × 10^−35^	2.2 × 10^−34^	0.048062	1.72 × 10^−38^	1.08 × 10^−8^	4.2 × 10^−95^
	Median	2.463873	0.130114	2.33 × 10^−8^	4.37 × 10^−35^	6.38 × 10^−35^	0.545056	8.26 × 10^−41^	3.18 × 10^−11^	5.81 × 10^−96^
	Rank	9	7	6	3	4	8	2	5	1
$F_{3}$	Mean	1536.896	589.492	279.3439	7.01 × 10^−15^	7.41 × 10^−15^	9.94 × 10^−8^	3.2 × 10^−19^	0.377007	9.93 × 10^−54^
	Best	1014.689	1.614937	81.91242	1.21 × 10^−16^	4.75 × 10^−20^	1.74 × 10^−12^	7.29 × 10^−30^	0.032038	2.74 × 10^−61^
	Worst	2165.455	5042.895	410.2312	5.57 × 10^−14^	7.75 × 10^−14^	1.74 × 10^−6^	3.9 × 10^−18^	0.687394	1.64 × 10^−52^
	Std	367.1974	1524.007	112.3045	1.27 × 10^−14^	1.9 × 10^−14^	3.87 × 10^−7^	9.9 × 10^−19^	0.201752	3.7 × 10^−53^
	median	1510.715	54.15445	291.4308	1.86 × 10^−15^	1.59 × 10^−16^	1.74 × 10^−8^	9.81 × 10^−21^	0.378658	7 × 10^−57^
	Rank	9	8	7	3	4	5	2	6	1
$F_{4}$	Mean	2.094247	3.963425	3.25 × 10^−9^	1.58 × 10^−15^	1.26 × 10^−14^	5.1 × 10^−5^	2.01 × 10^−22^	3.66 × 10^−8^	5.62 × 10^−79^
	Best	1.389849	1.60441	2.09 × 10^−9^	6.41 × 10^−16^	3.43 × 10^−16^	7.34 × 10^−6^	1.87 × 10^−52^	3.42 × 10^−17^	5.66 × 10^−80^
	Worst	3.003165	9.974081	4.66 × 10^−9^	3.25 × 10^−15^	1.08 × 10^−13^	0.000271	2.54 × 10^−21^	3.03 × 10^−7^	1.81 × 10^−78^
	Std	0.336995	2.204083	7.5 × 10^−10^	7.14 × 10^−16^	2.32 × 10^−14^	5.74 × 10^−5^	5.96 × 10^−22^	6.45 × 10^−8^	5.11 × 10^−79^
	Median	2.09854	3.260672	3.34 × 10^−9^	1.54 × 10^−15^	7.3 × 10^−15^	3.45 × 10^−5^	3.13 × 10^−27^	3.03 × 10^−8^	3.75 × 10^−79^
	Rank	8	9	5	3	4	7	2	6	1
$F_{5}$	Mean	310.4273	50.26246	36.10695	145.6653	26.8607	41.1588	28.76716	42.49733	25.30053
	Best	160.5013	3.647051	25.83811	120.7932	25.21201	39.3088	28.53831	41.58682	23.98133
	Worst	643.4969	150.2438	157.7053	188.3431	28.74824	41.3088	29.53865	43.53201	25.91348
	Std	120.443	36.5234	32.4626	19.73992	0.88407	0.48936	0.364848	0.615238	0.547028
	Median	279.5174	28.69298	26.07475	142.8936	26.70874	41.3088	28.53913	42.49068	25.41701
	Rank	9	7	4	8	2	5	3	6	1
$F_{6}$	Mean	14.55	20.25	0	0.45	0.642325	2.53 × 10^−9^	3.84 × 10^−20^	0.390869	0
	Best	6	5	0	0	1.57 × 10^−5^	1.95 × 10^−15^	6.74 × 10^−26^	0.274582	0
	Worst	35	46	0	1	1.25145	1.95 × 10^−8^	6.74 × 10^−19^	0.512766	0
	Std	5.835238	12.77281	0	0.510418	0.301075	4.05 × 10^−9^	1.5 × 10^−19^	0.080283	0
	Median	13.5	19	0	0	0.621487	1.95 × 10^−9^	6.74 × 10^−21^	0.406648	0
	Rank	7	8	1	5	6	3	2	4	1
$F_{7}$	Mean	0.00568	0.113413	0.020692	0.00313	0.000819	0.01946	0.000476	0.002182	0.000332
	Best	0.002111	0.029593	0.01006	0.001362	0.000248	0.002027	0.000105	0.001429	0.000104
	Worst	0.009546	0.202264	0.053628	0.006199	0.002048	0.021272	0.000473	0.002904	0.000647
	Std	0.002433	0.045866	0.01136	0.001351	0.000503	0.004115	0.000523	0.000466	0.000166
	Median	0.005365	0.107872	0.016995	0.002912	0.000629	0.020272	0.000405	0.00218	0.000376
	Rank	6	9	8	5	3	7	2	4	1
Sum rank		57	56	37	31	26	42	15	36	7
Mean rank		8.14	8	5.28	4.42	3.71	6	2.14	5.14	1
Total rank		9	8	6	4	3	7	2	5	1

**Table 3:** Optimization results for the indicated algorithm and unimodal functions.

		GA	PSO	GSA	TLBO	GWO	WOA	TSA	MPA	OOBO
$F_{8}$	Mean	−8184.41	−6908.66	−2849.07	−7803.6	−5885.12	−1633.58	−5669.65	−3652.14	−9285.56
	Best	−9717.68	−8501.44	−3969.23	−9103.77	−7227.05	−2358.57	−5706.3	−4419.9	−9378.27
	Worst	−6759.56	−4692.03	−2089.14	−5635.17	−3165.99	−1101.28	−5638.13	−2963.87	−5593.88
	Std	795.1373	836.7298	540.4078	986.7215	984.522	374.5959	21.89423	474.5819	16.428
	Median	−8117.66	−7098.95	−2671.33	−7735.22	−5774.63	−1649.72	−5669.63	−3632.84	−8697.73
	Rank	2	4	8	3	5	9	6	7	1
$F_{9}$	Mean	62.41143	57.06136	16.26758	10.67752	8.53 × 10^−15^	3.66599	0.005887	152.6917	0
	Best	36.86623	27.85883	4.974795	9.873963	0	1.78099	0.004776	128.2306	0
	Worst	89.88565	81.58644	25.86893	10.91936	5.68 × 10^−14^	6.78099	0.007215	177.2624	0
	Std	15.21578	16.51755	4.658667	0.397147	2.08 × 10^−14^	1.071779	0.000696	15.18171	0
	Median	61.67858	55.22468	15.42187	10.88657	0	3.78099	0.005871	154.6214	0
	Rank	8	7	6	5	2	4	3	9	1
$F_{10}$	Mean	3.221828	2.154679	3.57 × 10^−9^	0.263206	1.71 × 10^−14^	0.279159	6.38 × 10^−11^	8.31 × 10^−10^	6.04 × 10^−15^
	Best	2.757203	1.155151	2.64 × 10^−9^	0.156305	1.51 × 10^−14^	0.013128	8.14 × 10^−15^	1.68 × 10^−18^	4.44 × 10^−15^
	Worst	3.991866	3.403652	4.47 × 10^−9^	0.407323	2.22 × 10^−14^	0.612835	1.16 × 10^−9^	1.25 × 10^−8^	7.99 × 10^−15^
	Std	0.361776	0.549453	5.27 × 10^−10^	0.072866	3.15 × 10^−15^	0.146961	2.6 × 10^−10^	2.8 × 10^−9^	1.81 × 10^−15^
	Median	3.120322	2.170083	3.64 × 10^−9^	0.261541	1.51 × 10^−14^	0.312835	1.1 × 10^−13^	1.05 × 10^−11^	4.44 × 10^−15^
	Rank	9	8	5	6	2	7	3	4	1
$F_{11}$	Mean	1.230208	0.046292	3.737565	0.587684	0.003753	0.105701	1.55 × 10^−6^	0	0
	Best	1.140471	7.29 × 10^−9^	1.519288	0.310117	0	0.08107	4.23 × 10^−15^	0	0
	Worst	1.360027	0.166369	9.424268	0.900043	0.023851	0.11701	1.58 × 10^−5^	0	0
	Std	0.062759	0.051834	1.670291	0.169119	0.007344	0.007345	3.38 × 10^−6^	0	0
	Median	1.227231	0.029473	3.424268	0.582026	0	0.10701	8.77 × 10^−7^	0	0
	Rank	7	4	8	6	3	5	2	1	1
$F_{12}$	Mean	0.047026	0.480667	0.036283	0.020551	0.037211	1.55773	0.050163	0.082558	1.6 × 10^−7^
	Best	0.018364	0.000145	5.57 × 10^−3^	0.002031	0.019294	0.56726	0.035428	0.077912	2.87 × 10^−8^
	Worst	0.14047	2.089776	0.207317	0.137848	0.060775	2.56726	0.064276	0.086784	6.4 × 10^−7^
	Std	0.028483	0.602574	0.060865	0.028645	0.013876	0.4596	0.009855	0.002386	1.47 × 10^−7^
	Median	0.04179	0.1556	1.48 × 10^−2^	0.015181	0.032991	1.56726	0.050935	0.082108	1.08 × 10^−7^
	Rank	5	8	3	2	4	9	6	7	1
$F_{13}$	Mean	1.208544	0.508412	0.002085	0.329121	0.576319	0.338388	2.65875	0.565249	3.34 × 10^−4^
	Best	0.49809	0.099237	1.18 × 10^−3^	0.038266	0.297822	0.332688	2.63175	0.280295	1.38 × 10^−5^
	Worst	1.931337	5.497719	0.021024	0.790798	0.986896	0.338688	2.67175	0.863449	0.196466
	Std	0.333755	1.251681	0.005476	0.19894	0.170348	0.001342	0.009787	0.187819	0.061816
	Median	1.218053	0.043997	0.014381	0.282764	0.578323	0.338688	2.66175	0.579854	0.004399
	Rank	8	5	2	3	7	4	9	6	1
Sum rank		39	36	32	25	23	38	29	34	6
Mean rank		6.50	6	5.33	4.16	3.83	6.33	4.83	5.66	1
Total rank		9	7	5	3	2	8	4	6	1

**Table 4:** Optimization results for the indicated algorithm and unimodal functions.

		GA	PSO	GSA	TLBO	GWO	WOA	TSA	MPA	OOBO
$F_{14}$	Mean	0.99866	2.173587	3.59139	2.264278	3.740841	0.99823	1.798682	0.99875	0.9980
	Best	0.998004	0.998004	0.999508	0.998391	0.998004	0.998	0.998	0.998	0.9980
	Worst	1.009117	13.61861	8.906334	5.326656	12.67051	0.998004	2.912608	0.998	0.9980
	Std	0.002472	2.936539	2.778749	1.149633	3.969733	0.000272	0.527497	0.000328	0
	Median	0.998018	0.998004	2.986658	2.275231	2.982105	0.998104	1.912608	0.9983	0.9980
	Rank	3	6	8	7	9	2	5	4	1
$F_{\begin{array}{c} 15 \end{array}}$	Mean	0.005395	0.001684	0.002402	0.003169	0.00637	0.003719	0.000408	0.003936	0.000307
	Best	0.000775	0.000307	0.000805	0.002206	0.000307	0.000441	0.000364	0.003271	0.000307
	Worst	0.026587	0.022553	0.007021	0.003743	0.020363	0.00441	0.000532	0.0227	0.000307
	Std	0.008099	0.004932	0.001195	0.000394	0.009401	0.001248	7.59 × 10^−5^	0.005051	3.08 × 10^−15^
	Median	0.002074	0.000307	0.002311	0.003185	0.000308	0.00441	0.00039	0.0027	0.000307
	Rank	8	3	4	5	9	6	2	7	1
$F_{\begin{array}{c} 16 \end{array}}$	Mean	−1.03161	−1.03163	−1.03163	−1.03163	−1.03163	−1.0316	−1.0316	−1.03159	−1.03163
	Best	−1.03163	−1.03163	−1.03163	−1.03163	−1.03163	−1.0316	−1.03161	−1.0316	−1.03163
	Worst	−1.03147	−1.03163	−1.03163	−1.03163	−1.03163	−1.0316	−1.03158	−1.0315	−1.03163
	Std	3.5 × 10^−5^	1.35 × 10^−16^	1.76 × 10^−16^	2.28 × 10^−16^	8.38 × 10^−9^	3.66 × 10^−15^	8.67 × 10^−6^	3.06 × 1010^−5^	1.25 × 10^−16^
	Median	−1.03163	−1.03163	−1.03163	−1.03163	−1.03163	−1.0316	−1.0316	−1.0316	−1.03163
	Rank	2	1	1	1	1	3	3	4	1
$F_{17}$	Mean	0.436968	0.785443	0.397887	0.397887	0.397888	0.405051	0.400089	0.399298	0.397887
	Best	0.397888	0.397887	0.397887	0.397887	0.397887	0.399405	0.398052	0.39757	0.397887
	Worst	1.014779	2.791184	0.397887	0.397887	0.397889	0.41466	0.419052	0.40782	0.397887
	Std	0.140746	0.721755	3.17 × 10^−11^	7.06 × 10^−14^	4.5 × 10^−7^	0.00366	0.004481	0.003674	0
	Median	0.397897	0.397887	0.397887	0.397887	0.397888	0.40466	0.399052	0.39782	0.397887
	Rank	6	7	1	1	2	5	4	3	1
$F_{18}$	Mean	4.359299	3	3	3	3.000011	3.0001	3	3	3
	Best	3.000001	3	3	3	3	3	3	3	3
	Worst	30.00001	3	3	3	3.000038	3.000024	3	3	3
	Std	6.03523	2.64 × 10^−15^	1.8 × 10^−15^	6.28 × 10^−16^	1.06 × 10^−5^	3.50 × 10^−15^	8.19 × 10^−15^	6.31 × 10^−15^	0
	Median	3.001083	3	3	3	3.000006	3.0010	3.0002	3.0004	3
	Rank	4	1	1	1	2	3	1	1	1
$F_{19}$	Mean	−3.85434	−3.86278	−3.86278	−3.86138	−3.86217	−3.86166	−3.8066	−3.8627	−3.86278
	Best	−3.86278	−3.86278	−3.86278	−3.8625	−3.86278	−3.86276	−3.8366	−3.8627	−3.86278
	Worst	−3.81218	−3.86278	−3.86278	−3.85728	−3.8556	−3.85266	−3.7566	−3.8627	−3.86278
	Std	0.01484	2.07 × 10^−15^	3.92 × 10^−15^	0.001351	0.001696	0.003078	0.015218	2.38 × 1010^−15^	2.02 × 10^−15^
	Median	−3.86239	−3.86278	−3.86278	−3.862	−3.86276	−3.86266	−3.8066	−3.8627	−3.86278
	Rank	6	1	1	5	3	4	7	2	1
$F_{20}$	Mean	−2.8239	−3.26195	−3.3189	−3.20117	−3.25239	−3.23229	−3.31952	−3.3211	−3.322
	Best	−3.31342	−3.322	−3.322	−3.26174	−3.32199	−3.31342	−3.3212	−3.3213	−3.322
	Worst	−2.01325	−3.13764	−3.322	−3.12282	−3.08405	−3.13073	−3.3106	−3.32081	−3.322
	Std	0.385979	0.070639	4.56 × 10^−16^	0.031799	0.076571	0.035666	0.003085	8.35 × 1010^−5^	4.08 × 10^−16^
	Median	−2.96828	−3.322	−3.3170	−3.2076	−3.26248	−3.2424	−3.3206	−3.3211	−3.322
	Rank	9	5	4	8	6	7	3	2	1
$F_{21}$	Mean	−4.30401	−5.3892	−5.14867	−9.19017	−9.64524	−7.40509	−5.40209	−9.95445	−10.1532
	Best	−7.82781	−10.1532	−10.1532	−9.66387	−10.1532	−7.48159	−7.50209	−10.1532	−10.1532
	Worst	−2.10528	−2.63047	−2.68286	−9.1332	−5.05519	−7.32159	−3.50209	−8.15319	−10.1532
	Std	1.740823	3.019724	3.054624	0.120793	1.56199	0.033447	0.967906	0.532616	2.98 × 10^−9^
	Median	−4.16238	−5.10077	−3.64802	−9.1532	−10.1526	−7.40159	−5.50209	−10.1532	−10.1532
	Rank	9	7	8	4	3	5	6	2	1
$F_{22}$	Mean	−5.11742	−7.63234	−10.0847	−10.0487	−10.4025	−8.69973	−5.91349	−10.2859	−10.4029
	Best	−9.11064	−10.4029	−10.4029	−10.4029	−10.4028	−10.4029	−9.06249	−10.4029	−10.4029
	Worst	−2.6048	−2.7659	−4.03838	−9.08663	−10.402	−5.06249	−2.06249	−9.63378	−10.4029
	Std	1.969655	3.541736	1.423159	0.398279	0.000176	1.356185	1.754939	0.245412	6.32 × 10^−7^
	Median	−5.02966	−10.4029	−10.4029	−10.1836	−10.4025	−8.81649	−5.06249	−10.4029	−10.4029
	Rank	9	7	4	5	2	6	8	3	1
$F_{23}$	Mean	−6.56216	−6.1648	−10.5364	−9.26428	−10.1303	−10.0217	−9.80986	−10.1409	−10.5364
	Best	−10.2227	−10.5364	−10.5364	−10.534	−10.5363	−10.5364	−10.3683	−10.5364	−10.5364
	Worst	−2.79156	−2.42173	−10.5364	−3.50367	−2.42173	−9.36129	−3.36129	−5.53639	−10.5364
	Std	2.617323	3.734937	2.04 × 10^−15^	1.676539	1.814403	0.355819	1.606459	1.140168	2.61 × 10^−16^
	Median	−6.5629	−4.50554	−10.5364	−9.67172	−10.536	−10.0003	−10.3613	−10.5364	−10.5364
	Rank	7	8	1	6	3	4	5	2	1
Sum rank		63	46	33	43	40	45	44	30	10
Mean rank		6.30	4.60	3.30	4.30	4	4.50	4.40	3	1
Total rank		9	8	3	5	4	7	6	2	1

**Table 5:** Evaluation results of the sensitivity analysis of the OOBO algorithm in relation to *N*.

OF	Number of Population Members									
	10					20				
	Mean	Best	Worst	Std	Median	Mean	Best	Worst	Std	Median
$F_{1}$	1.5 × 10^−191^	6.7 × 10^−198^	1.2 × 10^−190^	0	4.6 × 10^−194^	6.9 × 10^−187^	5.4 × 10^−190^	5 × 10^−186^	0	1.1 × 10^−187^
$F_{2}$	2.6 × 10^−100^	6.9 × 10^−103^	2.2 × 10^−99^	5.4 × 10^−100^	2 × 10^−101^	1.12 × 10^−96^	3.86 × 10^−98^	7.36 × 10^−96^	1.65 × 10^−96^	5.98 × 10^−97^
$F_{3}$	4.18 × 10^−62^	1.17 × 10^−77^	5.75 × 10^−61^	1.36 × 10^−61^	7.35 × 10^−67^	2.06 × 10^−57^	9.28 × 10^−67^	2.49 × 10^−56^	5.98 × 10^−57^	7.83 × 10^−61^
$F_{4}$	2.23 × 10^−82^	4.84 × 10^−85^	3.61 × 10^−81^	8.03 × 10^−82^	9.93 × 10^−84^	9.96 × 10^−80^	3.6 × 10^−81^	5.46 × 10^−79^	1.6 × 10^−79^	2.19 × 10^−80^
$F_{5}$	27.09502	26.08308	27.99745	0.578805	27.07844	25.91036	25.41712	26.5326	0.275372	25.8901
$F_{6}$	0.05	0	1	0.223607	0	0	0	0	0	0
$F_{7}$	0.000496	4.76 × 10^−5^	0.001361	0.000317	0.000415	0.000337	5.88 × 10^−5^	0.000772	0.000226	0.000292
$F_{8}$	−6881.16	−8089.9	−4573.1	978.1824	−7191.94	−8016.92	−9258.33	−6741.5	651.0099	−8099.07
$F_{9}$	2.84 × 10^−15^	0	5.68 × 10^−14^	1.27 × 10^−14^	0	0	0	0	0	0
$F_{10}$	6.93 × 10^−15^	4.44 × 10^−15^	1.51 × 10^−14^	2.6 × 10^−15^	7.99 × 10^−15^	6.39 × 10^−15^	4.44 × 10^−15^	7.99 × 10^−15^	1.81 × 10^−15^	7.99 × 10^−15^
$F_{11}$	5.55 × 10^−18^	0	1.11 × 10^−16^	2.48 × 10^−17^	0	0	0	0	0	0
$F_{12}$	0.00674	8.22 × 10^−5^	0.048553	0.010942	0.002564	7.12 × 10^−5^	3.99 × 10^−7^	0.000814	0.000188	8.14 × 10^−6^
$F_{13}$	1.136331	0.46463	2.197278	0.437498	1.145889	0.195967	0.012237	0.787699	0.200471	0.143648
$F_{14}$	1.14691	0.998004	2.982105	0.485651	0.998004	1.047705	0.998004	1.992031	0.222271	0.998004
$F_{15}$	0.001316	0.000307	0.020363	0.004483	0.000307	0.000307	0.000307	0.000307	2.46 × 10^−13^	0.000307
$F_{16}$	−1.03163	−1.03163	−1.03163	7.2 × 10^−17^	−1.03163	−1.03163	−1.03163	−1.03163	1.76 × 10^−16^	−1.03163
$F_{17}$	0.397887	0.397887	0.397887	0	0.397887	0.397887	0.397887	0.397887	0	0.397887
$F_{18}$	3	3	3	8.52 × 10^−16^	3	3	3	3	3.67 × 10^−16^	3
$F_{19}$	−3.86278	−3.86278	−3.86278	1.78 × 10^−15^	−3.86278	−3.86278	−3.86278	−3.86278	1.96 × 10^−15^	−3.86278
$F_{20}$	−3.30416	−3.322	−3.2031	0.043556	−3.322	−3.322	−3.322	−3.322	3.67 × 10^−16^	−3.322
$F_{21}$	−8.67094	−10.1532	−2.63047	2.508958	−10.1532	−9.64336	−10.1532	−5.05448	1.569242	−10.1532
$F_{22}$	−8.94873	−10.4029	−3.7243	2.603594	−10.4029	−9.28095	−10.4029	−4.27237	2.307392	−10.4029
$F_{23}$	−8.91416	−10.5364	−2.87114	2.747411	−10.5364	−10.1913	−10.5364	−3.63468	1.543274	−10.5364
**OF**	**Number of Population Members**									
	**30**					**100**				
	Mean	Best	Worst	Std	Median	Mean	Best	Worst	Std	Median
$F_{1}$	3.90 × 10^−185^	2.30 × 10^−188^	3.10 × 10^−184^	0	4.40 × 10^−186^	4.1 × 10^−184^	8.4 × 10^−186^	3.6 × 10^−183^	0	2.2 × 10^−184^
$F_{2}$	1.94 × 10^−95^	5.82 × 10^−97^	1.93 × 10^−94^	4.20 × 10^−95^	5.81 × 10^−96^	5.29 × 10^−94^	1.12 × 10^−94^	1.42 × 10^−93^	3.39 × 10^−94^	4.03 × 10^−94^
$F_{3}$	9.93 × 10^−54^	2.74 × 10^−61^	1.64 × 10^−52^	3.70 × 10^−53^	7.00 × 10^−57^	3.23 × 10^−50^	5.39 × 10^−57^	4.14 × 10^−49^	9.3 × 10^−50^	2.68 × 10^−53^
$F_{4}$	5.62 × 10^−79^	5.66 × 10^−80^	1.81 × 10^−78^	5.11 × 10^−79^	3.75 × 10^−79^	4.59 × 10^−78^	1.86 × 10^−78^	1.21 × 10^−77^	2.3 × 10^−78^	3.97 × 10^−78^
$F_{5}$	25.30053	23.98133	25.91348	0.547028	25.41701	23.85676	23.27382	24.45052	0.338677	23.89411
$F_{6}$	0	0	0	0	0	0	0	0	0	0
$F_{7}$	0.000332	0.000104	0.000647	0.000166	0.000376	0.000132	3.66 × 10^−5^	0.000248	6.57 × 10^−5^	0.000139
$F_{8}$	−9285.56	−9378.27	−5593.88	16.428	−8697.73	−9287.85	−9631.41	−9033.35	207.1828	−9266.23
$F_{9}$	0	0	0	0	0	0	0	0	0	0
$F_{10}$	6.04 × 10^−15^	4.44 × 10^−15^	7.99 × 10^−15^	1.81 × 10^−15^	4.44 × 10^−15^	5.51 × 10^−15^	4.44 × 10^−15^	7.99 × 10^−15^	1.67 × 10^−15^	4.44 × 10^−15^
$F_{11}$	0	0	0	0	0	0	0	0	0	0
$F_{12}$	1.60 × 10^−7^	2.87 × 10^−8^	6.40 × 10^−7^	1.47 × 10^−7^	1.08 × 10^−7^	6.25 × 10^−11^	7.88 × 10^−12^	2 × 10^−10^	5.21 × 10^−11^	4.84 × 10^−11^
$F_{13}$	0.000334	1.38 × 10^−5^	0.196466	0.061816	0.004399	0.021178	1.13 × 10^−9^	0.109867	0.025469	0.010987
$F_{14}$	0.998	0.998	0.998	0	0.998	0.998004	0.998004	0.998004	0	0.998004
$F_{15}$	0.000307	0.000307	0.000307	3.08 × 10^−15^	0.000307	0.000307	0.000307	0.000307	4.44 × 10^−18^	0.000307
$F_{16}$	−1.03163	−1.03163	−1.03163	1.25 × 10^−16^	−1.03163	−1.03163	−1.03163	−1.03163	2.22 × 10^−16^	−1.03163
$F_{17}$	0.397887	0.397887	0.397887	0	0.397887	0.397887	0.397887	0.397887	0	0.397887
$F_{18}$	3	3	3	0	3	3	3	3	8.28 × 10^−16^	3
$F_{19}$	−3.86278	−3.86278	−3.86278	2.02 × 10^−15^	−3.86278	−3.86278	−3.86278	−3.86278	2.17 × 10^−15^	−3.86278
$F_{20}$	−3.322	−3.322	−3.322	4.08 × 10^−16^	−3.322	−3.322	−3.322	−3.322	4.56 × 10^−16^	−3.322
$F_{21}$	−10.1532	−10.1532	−10.1532	2.98 × 10^−9^	−10.1532	−10.1532	−10.1532	−10.1532	2.41 × 10^−15^	−10.1532
$F_{22}$	−10.4029	−10.4029	−10.4029	6.32 × 10^−7^	−10.4029	−10.4029	−10.4029	−10.4029	3.29 × 10^−15^	−10.4029
$F_{23}$	−10.5364	−10.5364	−10.5364	2.61 × 10^−16^	−10.5364	−10.5364	−10.5364	−10.5364	1.82 × 10^−15^	−10.5364

**Table 6:** Evaluation results of the sensitivity analysis of the OOBO algorithm in relation to *T*.

OF	Maximum Number of Iterations									
	200					500				
	Mean	Best	Worst	Std	Median	Mean	Best	Worst	Std	Median
$F_{1}$	7.17 × 10^−34^	1.03 × 10^−34^	2.57 × 10^−33^	6.11 × 10^−34^	4.15 × 10^−34^	3.93 × 10^−90^	1.58 × 10^−92^	6.23 × 10^−89^	1.38 × 10^−89^	3.47 × 10^−91^
$F_{2}$	4.09 × 10^−18^	2.34 × 10^−18^	6.97 × 10^−18^	1.55 × 10^−18^	3.6 × 10^−18^	5.9 × 10^−47^	7.18 × 10^−48^	3.64 × 10^−46^	7.74 × 10^−47^	3.76 × 10^−47^
$F_{3}$	8.81 × 10^−7^	3.15 × 10^−9^	1.48 × 10^−5^	3.3 × 10^−6^	2.17 × 10^−8^	1.11 × 10^−25^	5.92 × 10^−30^	1.6 × 10^−24^	3.59 × 10^−25^	7.49 × 10^−27^
$F_{4}$	1.18 × 10^−14^	4.13 × 10^−15^	2.62 × 10^−14^	6.31 × 10^−15^	9.69 × 10^−15^	1.09 × 10^−38^	1.98 × 10^−39^	3.21 × 10^−38^	9.58 × 10^−39^	8.38 × 10^−39^
$F_{5}$	27.68335	27.07442	28.4891	0.330926	27.67321	26.62557	25.92576	27.08981	0.364042	26.70357
$F_{6}$	0	0	0	0	0	0	0	0	0	0
$F_{7}$	0.001413	0.000531	0.002847	0.000596	0.001328	0.000698	0.000155	0.001848	0.000451	0.000613
$F_{8}$	−4205.53	−5420.94	−3509.74	446.4904	−4152.7	−5839.4	−7881.29	−4564.72	931.7564	−5730.07
$F_{9}$	0	0	0	0	0	0	0	0	0	0
$F_{10}$	6.04 × 10^−15^	4.44 × 10^−15^	7.99 × 10^−15^	1.81 × 10^−15^	4.44 × 10^−15^	6.22 × 10^−15^	4.44 × 10^−15^	7.99 × 10^−15^	1.82 × 10^−15^	6.22 × 10^−15^
$F_{11}$	0	0	0	0	0	0	0	0	0	0
$F_{12}$	0.016752	0.009609	0.036011	0.006497	0.015423	0.000514	3.29 × 10^−5^	0.004927	0.001084	0.000143
$F_{13}$	0.858035	0.595632	1.12689	0.166997	0.839516	0.283433	0.020638	0.790443	0.228379	0.197344
$F_{14}$	1.017961	0.998004	1.396217	0.089032	0.998004	0.998004	0.998004	0.998004	1.25 × 10^−16^	0.998004
$F_{15}$	0.000375	0.000314	0.000482	6.23 × 10^−5^	0.000343	0.000308	0.000307	0.000309	3.19 × 10^−7^	0.000308
$F_{16}$	−1.03163	−1.03163	−1.03163	1.02 × 10^−16^	−1.03163	−1.03163	−1.03163	−1.03163	1.53 × 10^−16^	−1.03163
$F_{17}$	0.397887	0.397887	0.397887	0	0.397887	0.397887	0.397887	0.397887	0	0.397887
$F_{18}$	3	3	3	1.06 × 10^−15^	3	3	3	3	1.48 × 10^−15^	3
$F_{19}$	−3.86278	−3.86278	−3.86278	1.92 × 10^−15^	−3.86278	−3.86278	−3.86278	−3.86278	1.99 × 10^−15^	−3.86278
$F_{20}$	−3.32183	−3.322	−3.31867	0.000743	−3.322	−3.322	−3.322	−3.322	8.82 × 10^−12^	−3.322
$F_{21}$	−9.87108	−10.1532	−5.11428	1.123381	−10.1532	−10.1452	−10.1532	−9.99344	0.035723	−10.1532
$F_{22}$	−9.79396	−10.4029	−5.08767	1.592579	−10.4029	−9.87141	−10.4029	−5.08767	1.636005	−10.4029
$F_{23}$	−10.3874	−10.5364	−7.55668	0.666275	−10.5364	−10.5191	−10.5364	−10.19	0.07746	−10.5364
**OF**	**Maximum Number of Iterations**									
	**800**					**1000**				
	Mean	Best	Worst	Std	Median	Mean	Best	Worst	Std	Median
$F_{1}$	1.4 × 10^−147^	1 × 10^−149^	9.1 × 10^−147^	2.4 × 10^−147^	5.4 × 10^−148^	3.90 × 10^−185^	2.30 × 10^−188^	3.10 × 10^−184^	0	4.40 × 10^−186^
$F_{2}$	4.23 × 10^−76^	2.88 × 10^−77^	1.29 × 10^−75^	3.62 × 10^−76^	3.95 × 10^−76^	1.94 × 10^−95^	5.82 × 10^−97^	1.93 × 10^−94^	4.20 × 10^−95^	5.81 × 10^−96^
$F_{3}$	1.6 × 10^−43^	2.96 × 10^−50^	2.93 × 10^−42^	6.54 × 10^−43^	5.78 × 10^−46^	9.93 × 10^−54^	2.74 × 10^−61^	1.64 × 10^−52^	3.70 × 10^−53^	7.00 × 10^−57^
$F_{4}$	6.4 × 10^−63^	9.79 × 10^−64^	2.12 × 10^−62^	5.17 × 10^−63^	5.4 × 10^−63^	5.62 × 10^−79^	5.66 × 10^−80^	1.81 × 10^−78^	5.11 × 10^−79^	3.75 × 10^−79^
$F_{5}$	25.56784	24.19419	26.68351	0.59798	25.61208	25.30053	23.98133	25.91348	0.547028	25.41701
$F_{6}$	0	0	0	0	0	0	0	0	0	0
$F_{7}$	0.000336	0.000105	0.000672	0.000148	0.000305	0.000332	0.000104	0.000647	0.000166	0.000376
$F_{8}$	−7158.54	−9016.07	−5204.52	1096.436	−7361.04	−9285.56	−9378.27	−5593.88	16.428	−8697.73
$F_{9}$	0	0	0	0	0	0	0	0	0	0
$F_{10}$	6.57 × 10^−15^	4.44 × 10^−15^	7.99 × 10^−15^	1.79 × 10^−15^	7.99 × 10^−15^	6.04 × 10^−15^	4.44 × 10^−15^	7.99 × 10^−15^	1.81 × 10^−15^	4.44 × 10^−15^
$F_{11}$	0	0	0	0	0	0	0	0	0	0
$F_{12}$	4.35 × 10^−6^	4.93 × 10^−7^	3.99 × 10^−5^	8.61 × 10^−6^	2.08 × 10^−6^	1.60 × 10^−7^	2.87 × 10^−8^	6.40 × 10^−7^	1.47 × 10^−7^	1.08 × 10^−7^
$F_{13}$	0.113476	0.000437	0.256611	0.0816	0.129648	0.000334	1.38 × 10^−5^	0.196466	0.061816	0.004399
$F_{14}$	0.998004	0.998004	0.998004	5.09 × 10^−17^	0.998004	0.998	0.998	0.998	0	0.998
$F_{15}$	0.000307	0.000307	0.000307	1.15 × 10^−9^	0.000307	0.000307	0.000307	0.000307	3.08 × 10^−15^	0.000307
$F_{16}$	−1.03163	−1.03163	−1.03163	1.69 × 10^−16^	−1.03163	−1.03163	−1.03163	−1.03163	1.25 × 10^−16^	−1.03163
$F_{17}$	0.397887	0.397887	0.397887	0	0.397887	0.397887	0.397887	0.397887	0	0.397887
$F_{18}$	3	3	3	5.67 × 10^−16^	3	3	3	3	0	3
$F_{19}$	−3.86278	−3.86278	−3.86278	1.85 × 10^−15^	−3.86278	−3.86278	−3.86278	−3.86278	2.02 × 10^−15^	−3.86278
$F_{20}$	−3.322	−3.322	−3.322	8.94 × 10^−16^	−3.322	−3.322	−3.322	−3.322	4.08 × 10^−16^	−3.322
$F_{21}$	−10.1525	−10.1532	−10.1463	0.002115	−10.1532	−10.1532	−10.1532	−10.1532	2.98 × 10^−9^	−10.1532
$F_{22}$	−10.4029	−10.4029	−10.4029	7.99 × 10^−15^	−10.4029	−10.4029	−10.4029	−10.4029	6.32 × 10^−7^	−10.4029
$F_{23}$	−10.5364	−10.5364	−10.5364	2.45 × 10^−12^	−10.5364	−10.5364	−10.5364	−10.5364	2.61 × 10^−16^	−10.5364

**Table 7:** Scalability study results of OOBO.

OF	Dimension									
	30					50				
	Mean	Best	Worst	Std	Median	Mean	Best	Worst	Std	Median
$F_{1}$	3.90 × 10^−185^	2.30 × 10^−188^	3.10 × 10^−184^	0	4.40 × 10^−186^	7.5 × 10^−181^	9.6 × 10^−183^	4.8 × 10^−180^	0	9.7 × 10^−182^
$F_{2}$	1.94 × 10^−95^	5.82 × 10^−97^	1.93 × 10^−94^	4.20 × 10^−95^	5.81 × 10^−96^	1.81 × 10^−93^	1.22 × 10^−94^	9.67 × 10^−93^	2.18 × 10^−93^	1.11 × 10^−93^
$F_{3}$	9.93 × 10^−54^	2.74 × 10^−61^	1.64 × 10^−52^	3.70 × 10^−53^	7.00 × 10^−57^	4.8 × 10^−42^	2.54 × 10^−52^	9.15 × 10^−41^	2.04 × 10^−41^	6.06 × 10^−48^
$F_{4}$	5.62 × 10^−79^	5.66 × 10^−80^	1.81 × 10^−78^	5.11 × 10^−79^	3.75 × 10^−79^	3.41 × 10^−75^	2.81 × 10^−76^	1.05 × 10^−74^	2.81 × 10^−75^	2.59 × 10^−75^
$F_{5}$	25.30053	23.98133	25.91348	0.547028	25.41701	45.95853	45.13515	46.76063	0.439077	46.02327
$F_{6}$	0	0	0	0	0	0	0	0	0	0
$F_{7}$	0.000332	0.000104	0.000647	0.000166	0.000376	0.000335	9.13 × 10^−5^	0.000704	0.000163	0.000322
$F_{8}$	−9285.56	−9378.27	−5593.88	16.428	−8697.73	−10671.4	−15108.4	−6633.51	19.907	−10913.9
$F_{9}$	0	0	0	0	0	0	0	0	0	0
$F_{10}$	6.04 × 10^−15^	4.44 × 10^−15^	7.99 × 10^−15^	1.81 × 10^−15^	4.44 × 10^−15^	6.39 × 10^−15^	4.44 × 10^−15^	7.99 × 10^−15^	1.81 × 10^−15^	7.99 × 10^−15^
$F_{11}$	0	0	0	0	0	0	0	0	0	0
$F_{12}$	1.60 × 10^−7^	2.87 × 10^−8^	6.40 × 10^−7^	1.47 × 10^−7^	1.08 × 10^−7^	0.000874	6.81 × 10^−5^	0.005473	0.001312	0.000327
$F_{13}$	0.000334	1.38 × 10^−5^	0.196466	0.061816	0.004399	0.927678	0.080005	2.719466	0.565395	0.957426
**OF**	**Dimension**									
	**80**					**100**				
	Mean	Best	Worst	Std	Median	Mean	Best	Worst	Std	Median
$F_{1}$	6.6 × 10^−178^	2.2 × 10^−180^	3 × 10^−177^	0	3 × 10^−178^	5.7 × 10^−177^	1.1 × 10^−179^	5.4 × 10^−176^	0	2.1 × 10^−177^
$F_{2}$	3.35 × 10^−92^	3.35 × 10^−93^	9.07 × 10^−92^	2.62 × 10^−92^	2.42 × 10^−92^	7.45 × 10^−92^	1.7 × 10^−92^	2.88 × 10^−91^	7.69 × 10^−92^	4.3 × 10^−92^
$F_{3}$	4.43 × 10^−35^	5.4 × 10^−48^	8.85 × 10^−34^	1.98 × 10^−34^	1.55 × 10^−40^	8.59 × 10^−30^	3.29 × 10^−41^	1.7 × 10^−28^	3.8 × 10^−29^	2.9 × 10^−36^
$F_{4}$	2.61 × 10^−73^	5.26 × 10^−74^	6.6 × 10^−73^	1.77 × 10^−73^	2.25 × 10^−73^	4.06 × 10^−72^	8.54 × 10^−74^	2.55 × 10^−71^	6.03 × 10^−72^	1.46 × 10^−72^
$F_{5}$	76.28851	75.48869	78.2779	0.614848	76.22384	96.93315	95.92653	98.14792	0.747228	96.69143
$F_{6}$	0	0	0	0	0	0	0	0	0	0
$F_{7}$	0.000304	0.000139	0.000702	0.00013	0.000299	0.000315	8.21 × 10^−5^	0.000679	0.000161	0.000306
$F_{8}$	−14110.3	−18685.9	−8549.06	3251.38	−14096.5	−16057.7	−21661.7	−9609.56	4037.696	−16138.5
$F_{9}$	0	0	0	0	0	0	0	0	0	0
$F_{10}$	6.93 × 10^−15^	4.44 × 10^−15^	7.99 × 10^−15^	1.67 × 10^−15^	7.99 × 10^−15^	7.28 × 10^−15^	4.44 × 10^−15^	7.99 × 10^−15^	1.46 × 10^−15^	7.99 × 10^−15^
$F_{11}$	0	0	0	0	0	0	0	0	0	0
$F_{12}$	0.017111	0.010001	0.043843	0.007843	0.014707	0.035716	0.019816	0.064286	0.010429	0.03414
$F_{13}$	6.26385	2.679753	7.919206	2.145142	7.579479	8.965042	4.672791	9.909291	1.857927	9.900987
**OF**	**Dimension**									
	**250**					**500**				
	Mean	Best	Worst	Std	Median	Mean	Best	Worst	Std	Median
$F_{1}$	1.5 × 10^−174^	3.4 × 10^−176^	1 × 10^−173^	0	8.1 × 10^−175^	9.1 × 10^−174^	4.7 × 10^−175^	7.3 × 10^−173^	0	2.3 × 10^−174^
$F_{2}$	1.06 × 10^−90^	2.13 × 10^−91^	4.15 × 10^−90^	9.33 × 10^−91^	7.04 × 10^−91^	5.46 × 10^−90^	1.07 × 10^−90^	2.08 × 10^−89^	4.82 × 10^−90^	3.28 × 10^−90^
$F_{3}$	3.42 × 10^−24^	1.39 × 10^−37^	6.78 × 10^−23^	1.51 × 10^−23^	2.31 × 10^−30^	1.58 × 10^−21^	6.28 × 10^−33^	2.55 × 10^−20^	5.76 × 10^−21^	2.01 × 10^−27^
$F_{4}$	1.02 × 10^−69^	1.05 × 10^−70^	2.8 × 10^−69^	7.8 × 10^−70^	8.34 × 10^−70^	2.51 × 10^−68^	5.1 × 10^−69^	1.26 × 10^−67^	3.08 × 10^−68^	1.53 × 10^−68^
$F_{5}$	247.2909	246.1068	247.7164	0.535183	247.5457	497.2267	495.61	497.4878	0.398995	497.3465
$F_{6}$	0	0	0	0	0	0	0	0	0	0
$F_{7}$	0.000452	0.000192	0.001046	0.000196	0.000426	0.000442	0.000166	0.000684	0.000153	0.000419
$F_{8}$	−26978.1	−43004	−15902.6	6959.373	−25886.1	−39583.8	−72865.8	−22536.4	13775.52	−40552.4
$F_{9}$	0	0	0	0	0	0	0	0	0	0
$F_{10}$	7.99 × 10^−15^	7.99 × 10^−15^	7.99 × 10^−15^	0	7.99 × 10^−15^	7.99 × 10^−15^	7.99 × 10^−15^	7.99 × 10^−15^	0	7.99 × 10^−15^
$F_{11}$	0	0	0	0	0	0	0	0	0	0
$F_{12}$	0.237375	0.204464	0.290841	0.025523	0.231674	0.518869	0.472406	0.571823	0.025782	0.518707
$F_{13}$	24.85032	24.84133	24.85882	0.004731	24.85006	49.81471	49.80007	49.83025	0.007964	49.81408

**Table 8:** Optimization results of the indicated algorithm and functions.

		OOBO	MPA	TSA	WOA	GWO	TLBO	GSA	PSO	GA
C17-F1	Mean	1.00 × 10^2^	3.48 × 10^3^	1.19 × 10^9^	6.55 × 10^6^	1.70 × 10^4^	1.41 × 10^8^	3.06 × 10^2^	3.01 × 10^3^	1.80 × 10^7^
	Best	1.00 × 10^2^	2.18 × 10^3^	1.05 × 10^7^	3.01 × 10^6^	1.05 × 10^4^	6.28 × 10^7^	1.08 × 10^2^	3.35 × 10^2^	5.99 × 10^6^
	Worst	1.00 × 10^2^	5.14 × 10^3^	3.41 × 10^9^	9.20 × 10^6^	2.53 × 10^4^	3.40 × 10^8^	6.81 × 10^2^	8.92 × 10^3^	3.29 × 10^7^
	Std	1.76 × 10^−5^	1.38 × 10^3^	1.52 × 10^9^	3.09 × 10^6^	6.91 × 10^3^	1.34 × 10^8^	2.61 × 10^2^	3.97 × 10^3^	1.11 × 10^7^
	Median	1.00 × 10^2^	3.31 × 10^3^	6.70 × 10^8^	7.00 × 10^6^	1.61 × 10^4^	8.05 × 10^7^	2.16 × 10^2^	1.40 × 10^3^	1.65 × 10^7^
	Rank	1	4	9	6	5	8	2	3	7
C17-F3	Mean	3.00 × 10^2^	8.18 × 10^2^	1.03 × 10^4^	9.32 × 10^2^	2.89 × 10^3^	7.08 × 10^2^	1.03 × 10^4^	3.00 × 10^2^	1.42 × 10^4^
	Best	3.00 × 10^2^	3.56 × 10^2^	6.55 × 10^3^	4.81 × 10^2^	5.80 × 10^2^	4.64 × 10^2^	8.39 × 10^3^	3.00 × 10^2^	4.18 × 10^3^
	Worst	3.00 × 10^2^	1.60 × 10^3^	1.38 × 10^4^	1.61 × 10^3^	6.78 × 10^3^	8.67 × 10^2^	1.16 × 10^4^	3.00 × 10^2^	2.24 × 10^4^
	Std	5.43 × 10^−11^	5.90 × 10^2^	2.97 × 10^3^	4.96 × 10^2^	2.95 × 10^3^	1.77 × 10^2^	1.40 × 10^3^	5.54 × 10−12	9.50 × 10^3^
	Median	3.00 × 10^2^	6.59 × 10^2^	1.04 × 10^4^	8.20 × 10^2^	2.09 × 10^3^	7.50 × 10^2^	1.05 × 10^4^	3.00 × 10^2^	1.50 × 10^4^
	Rank	2	4	8	5	6	3	7	1	9
C17-F4	Mean	4.00 × 10^2^	4.04 × 10^2^	5.64 × 10^2^	4.28 × 10^2^	4.21 × 10^2^	4.09 × 10^2^	4.06 × 10^2^	4.19 × 10^2^	4.14 × 10^2^
	Best	4.00 × 10^2^	4.01 × 10^2^	4.06 × 10^2^	4.06 × 10^2^	4.06 × 10^2^	4.08 × 10^2^	4.05 × 10^2^	4.00 × 10^2^	4.11 × 10^2^
	Worst	4.00 × 10^2^	4.13 × 10^2^	8.91 × 10^2^	4.51 × 10^2^	4.63 × 10^2^	4.09 × 10^2^	4.06 × 10^2^	4.67 × 10^2^	4.18 × 10^2^
	Std	4.02 × 10^−3^	5.65 × 10^0^	2.21 × 10^2^	2.45 × 10^1^	2.82 × 10^1^	5.26 × 10^−1^	7.02 × 10−01	3.23 × 10^1^	2.83 × 10^0^
	Median	4.00 × 10^2^	4.01 × 10^2^	4.79 × 10^2^	4.27 × 10^2^	4.07 × 10^2^	4.09 × 10^2^	4.06 × 10^2^	4.05 × 10^2^	4.14 × 10^2^
	Rank	1	2	9	8	7	4	3	6	5
C17-F5	Mean	5.10 × 10^2^	5.12 × 10^2^	5.65 × 10^2^	5.35 × 10^2^	5.17 × 10^2^	5.34 × 10^2^	5.49 × 10^2^	5.28 × 10^2^	5.28 × 10^2^
	Best	5.08 × 10^2^	5.10 × 10^2^	5.32 × 10^2^	5.14 × 10^2^	5.11 × 10^2^	5.28 × 10^2^	5.37 × 10^2^	5.12 × 10^2^	5.23 × 10^2^
	Worst	5.14 × 10^2^	5.13 × 10^2^	5.84 × 10^2^	5.57 × 10^2^	5.27 × 10^2^	5.37 × 10^2^	5.61 × 10^2^	5.51 × 10^2^	5.34 × 10^2^
	Std	2.55 × 10^0^	1.42 × 10	2.31 × 10^1^	1.77 × 10^1^	7.10 × 10^0^	3.85 × 10^0^	1.12 × 10^1^	1.80 × 10^1^	4.57 × 10^0^
	Median	5.09 × 10^2^	5.12 × 10^2^	5.72 × 10^2^	5.35 × 10^2^	5.16 × 10^2^	5.35 × 10^2^	5.49 × 10^2^	5.25 × 10^2^	5.28 × 10^2^
	Rank	1	2	9	7	3	6	8	4	5
C17-F6	Mean	6.00 × 10^2^	6.00 × 10^2^	6.25 × 10^2^	6.36 × 10^2^	6.01 × 10^2^	6.07 × 10^2^	6.17 × 10^2^	6.07 × 10^2^	6.10 × 10^2^
	Best	6.00 × 10^2^	6.00 × 10^2^	6.11 × 10^2^	6.31 × 10^2^	6.01 × 10^2^	6.05 × 10^2^	6.08 × 10^2^	6.01 × 10^2^	6.07 × 10^2^
	Worst	6.00 × 10^2^	6.01 × 10^2^	6.40 × 10^2^	6.46 × 10^2^	6.03 × 10^2^	6.10 × 10^2^	6.27 × 10^2^	6.19 × 10^2^	6.14 × 10^2^
	Std	1.33 × 10^−4^	1.78 × 10^−1^	1.20 × 10^1^	6.68 × 10^0^	8.90 × 10^−1^	2.38 × 10^0^	7.76 × 10^0^	7.89 × 10^0^	3.27 × 10^0^
	Median	6.00 × 10^2^	6.00 × 10^2^	6.24 × 10^2^	6.34 × 10^2^	6.01 × 10^2^	6.06 × 10^2^	6.16 × 10^2^	6.04 × 10^2^	6.10 × 10^2^
	Rank	1	2	8	9	3	4	7	5	6
C17-F7	Mean	7.20 × 10^2^	7.22 × 10^2^	8.13 × 10^2^	7.67 × 10^2^	7.29 × 10^2^	7.52 × 10^2^	7.17 × 10^2^	7.33 × 10^2^	7.37 × 10^2^
	Best	7.15 × 10^2^	7.19 × 10^2^	7.86 × 10^2^	7.47 × 10^2^	7.22 × 10^2^	7.47 × 10^2^	7.12 × 10^2^	7.26 × 10^2^	7.27 × 10^2^
	Worst	7.24 × 10^2^	7.24 × 10^2^	8.48 × 10^2^	7.94 × 10^2^	7.46 × 10^2^	7.60 × 10^2^	7.25 × 10^2^	7.44 × 10^2^	7.41 × 10^2^
	Std	3.77 × 10^0^	1.96 × 10^0^	2.58 × 10^1^	2.26 × 10^1^	1.15 × 10^1^	5.52 × 10^0^	5.59 × 10^0^	8.27 × 10^0^	6.53 × 10^0^
	Median	7.20 × 10^2^	7.22 × 10^2^	8.10 × 10^2^	7.64 × 10^2^	7.24 × 10^2^	7.50 × 10^2^	7.15 × 10^2^	7.31 × 10^2^	7.40 × 10^2^
	Rank	2	3	9	8	4	7	1	5	6
C17-F8	Mean	8.10 × 10^2^	8.11 × 10^2^	8.32 × 10^2^	8.36 × 10^2^	8.15 × 10^2^	8.38 × 10^2^	8.22 × 10^2^	8.23 × 10^2^	8.17 × 10^2^
	Best	8.08 × 10^2^	8.10 × 10^2^	8.12 × 10^2^	8.24 × 10^2^	8.10 × 10^2^	8.31 × 10^2^	8.16 × 10^2^	8.16 × 10^2^	8.13 × 10^2^
	Worst	8.13 × 10^2^	8.14 × 10^2^	8.52 × 10^2^	8.49 × 10^2^	8.21 × 10^2^	8.46 × 10^2^	8.30 × 10^2^	8.29 × 10^2^	8.25 × 10^2^
	Std	2.07 × 10^0^	1.60 × 10^0^	1.68 × 10^1^	1.03 × 10^1^	4.66 × 10^0^	7.60 × 10^0^	6.74 × 10^0^	6.39 × 10^0^	5.32 × 10^0^
	Median	8.10 × 10^2^	8.11 × 10^2^	8.31 × 10^2^	8.36 × 10^2^	8.14 × 10^2^	8.37 × 10^2^	8.22 × 10^2^	8.23 × 10^2^	8.15 × 10^2^
	Rank	1	2	7	8	3	9	5	6	4
C17-F9	Mean	9.00 × 10^2^	9.00 × 10^2^	1.23 × 10^3^	1.12 × 10^3^	9.01 × 10^2^	9.11 × 10^2^	9.00 × 10^2^	9.04 × 10^2^	9.05 × 10^2^
	Best	9.00 × 10^2^	9.00 × 10^2^	9.28 × 10^2^	9.95 × 10^2^	9.00 × 10^2^	9.07 × 10^2^	9.00 × 10^2^	9.01 × 10^2^	9.03 × 10^2^
	Worst	9.00 × 10^2^	9.00 × 10^2^	1.62 × 10^3^	1.40 × 10^3^	9.01 × 10^2^	9.19 × 10^2^	9.00 × 10^2^	9.12 × 10^2^	9.09 × 10^2^
	Std	6.63E −08	6.75E −02	3.02 × 10^2^	1.90 × 10^2^	3.38E −01	5.46 × 10^0^	6.78E −09	5.30 × 10^0^	2.76 × 10^0^
	Median	9.00 × 10^2^	9.00 × 10^2^	1.20 × 10^3^	1.04 × 10^3^	9.01 × 10^2^	9.10 × 10^2^	9.00 × 10^2^	9.02 × 10^2^	9.04 × 10^2^
	Rank	2	3	9	8	4	7	1	5	6
C17-F10	Mean	1.33 × 10^3^	1.37 × 10^3^	2.28 × 10^3^	2.35 × 10^3^	1.52 × 10^3^	2.16 × 10^3^	2.44 × 10^3^	1.94 × 10^3^	1.72 × 10^3^
	Best	1.15 × 10^3^	1.22 × 10^3^	2.09 × 10^3^	2.02 × 10^3^	1.41 × 10^3^	1.80 × 10^3^	2.05 × 10^3^	1.59 × 10^3^	1.41 × 10^3^
	Worst	1.47 × 10^3^	1.48 × 10^3^	2.64 × 10^3^	2.76 × 10^3^	1.68 × 10^3^	2.44 × 10^3^	2.73 × 10^3^	2.34 × 10^3^	2.11 × 10^3^
	Std	1.35 × 10^2^	1.11 × 10^2^	2.45 × 10^2^	3.09 × 10^2^	1.18 × 10^2^	2.74 × 10^2^	3.06 × 10^2^	3.10 × 10^2^	2.96 × 10^2^
	Median	1.36 × 10^3^	1.39 × 10^3^	2.19 × 10^3^	2.31 × 10^3^	1.49 × 10^3^	2.20 × 10^3^	2.49 × 10^3^	1.92 × 10^3^	1.68 × 10^3^
	Rank	1	2	7	8	3	6	9	5	4
C17-F11	Mean	1.10 × 10^3^	1.11 × 10^3^	3.21 × 10^3^	1.25 × 10^3^	1.13 × 10^3^	1.15 × 10^3^	1.13 × 10^3^	1.14 × 10^3^	2.33 × 10^3^
	Best	1.10 × 10^3^	1.10 × 10^3^	1.21 × 10^3^	1.13 × 10^3^	1.11 × 10^3^	1.14 × 10^3^	1.12 × 10^3^	1.13 × 10^3^	1.11 × 10^3^
	Worst	1.10 × 10^3^	1.11 × 10^3^	5.22 × 10^3^	1.43 × 10^3^	1.14 × 10^3^	1.17 × 10^3^	1.13 × 10^3^	1.16 × 10^3^	5.79 × 10^3^
	Std	1.47 × 10^0^	1.85 × 10^0^	2.23 × 10^3^	1.36 × 10^2^	1.19 × 10^1^	1.44 × 10^1^	6.29 × 10^0^	1.42 × 10^1^	2.31 × 10^3^
	Median	1.10 × 10^3^	1.11 × 10^3^	3.20 × 10^3^	1.23 × 10^3^	1.13 × 10^3^	1.15 × 10^3^	1.13 × 10^3^	1.14 × 10^3^	1.21 × 10^3^
	Rank	1	2	9	7	3	6	4	5	8
C17-F12	Mean	1.24 × 10^3^	2.74 × 10^5^	2.44 × 10^5^	7.46 × 10^6^	1.37 × 10^6^	4.87 × 10^6^	4.73 × 10^5^	7.84 × 10^3^	5.83 × 10^5^
	Best	1.20 × 10^3^	6.36 × 10^4^	8.09 × 10^4^	9.20 × 10^5^	3.13 × 10^5^	1.30 × 10^6^	7.75 × 10^4^	2.46 × 10^3^	1.69 × 10^5^
	Worst	1.32 × 10^3^	3.82 × 10^5^	3.29 × 10^5^	1.66 × 10^7^	1.91 × 10^6^	8.62 × 10^6^	1.04 × 10^6^	1.34 × 10^4^	1.03 × 10^6^
	Std	5.63 × 10^1^	1.47 × 10^5^	1.14 × 10^5^	6.59 × 10^6^	7.37 × 10^5^	3.88 × 10^6^	4.36 × 10^5^	5.01 × 10^3^	3.53 × 10^5^
	Median	1.21 × 10^3^	3.26 × 10^5^	2.82 × 10^5^	6.16 × 10^6^	1.63 × 10^6^	4.78 × 10^6^	3.85 × 10^5^	7.72 × 10^3^	5.67 × 10^5^
	Rank	1	4	3	9	7	8	5	2	6
C17-F13	Mean	1.30 × 10^3^	3.50 × 10^3^	6.45 × 10^3^	1.88 × 10^4^	1.23 × 10^4^	1.62 × 10^4^	1.06 × 10^4^	6.43 × 10^3^	5.25 × 10^4^
	Best	1.30 × 10^3^	1.38 × 10^3^	3.19 × 10^3^	7.46 × 10^3^	1.69 × 10^3^	1.53 × 10^4^	8.94 × 10^3^	2.34 × 10^3^	8.28 × 10^3^
	Worst	1.31 × 10^3^	6.31 × 10^3^	8.77 × 10^3^	3.02 × 10^4^	2.63 × 10^4^	1.84 × 10^4^	1.19 × 10^4^	1.62 × 10^4^	1.74 × 10^5^
	Std	3.22 × 10^0^	2.25 × 10^3^	2.74 × 10^3^	1.00 × 10^4^	1.13 × 10^4^	1.48 × 10^3^	1.25 × 10^3^	6.56 × 10^3^	8.08 × 10^4^
	Median	1.31 × 10^3^	3.16 × 10^3^	6.92 × 10^3^	1.88 × 10^4^	1.06 × 10^4^	1.55 × 10^4^	1.08 × 10^4^	3.61 × 10^3^	1.41 × 10^4^
	Rank	1	2	4	8	6	7	5	3	9
C17-F14	Mean	1.40 × 10^3^	1.54 × 10^3^	2.38 × 10^3^	1.94 × 10^3^	2.11 × 10^3^	1.58 × 10^3^	6.39 × 10^3^	2.94 × 10^3^	1.26 × 10^4^
	Best	1.40 × 10^3^	1.42 × 10^3^	1.48 × 10^3^	1.53 × 10^3^	1.50 × 10^3^	1.51 × 10^3^	3.76 × 10^3^	1.43 × 10^3^	3.65 × 10^3^
	Worst	1.40 × 10^3^	1.90 × 10^3^	5.01 × 10^3^	2.51 × 10^3^	3.90 × 10^3^	1.61 × 10^3^	8.59 × 10^3^	6.65 × 10^3^	2.50 × 10^4^
	Std	1.72 × 10^0^	2.39 × 10^2^	1.75 × 10^3^	4.10 × 10^2^	1.20 × 10^3^	4.84 × 10^1^	2.56 × 10^3^	2.50 × 10^3^	9.04 × 10^3^
	Median	1.40 × 10^3^	1.43 × 10^3^	1.52 × 10^3^	1.87 × 10^3^	1.52 × 10^3^	1.61 × 10^3^	6.61 × 10^3^	1.84 × 10^3^	1.08 × 10^4^
	Rank	1	2	6	4	5	3	8	7	9
C17-F15	Mean	1.50 × 10^3^	2.67 × 10^3^	7.41 × 10^3^	9.21 × 10^3^	7.36 × 10^3^	1.70 × 10^3^	2.04 × 10^4^	8.73 × 10^3^	4.44 × 10^3^
	Best	1.50 × 10^3^	1.52 × 10^3^	1.60 × 10^3^	2.23 × 10^3^	1.60 × 10^3^	1.58 × 10^3^	8.86 × 10^3^	2.82 × 10^3^	1.88 × 10^3^
	Worst	1.50 × 10^3^	3.69 × 10^3^	2.14 × 10^4^	1.71 × 10^4^	1.24 × 10^4^	1.79 × 10^3^	2.87 × 10^4^	1.43 × 10^4^	7.79 × 10^3^
	Std	4.93E −01	9.39 × 10^2^	9.41 × 10^3^	6.12 × 10^3^	4.69 × 10^3^	1.02 × 10^2^	9.61 × 10^3^	4.81 × 10^3^	2.94 × 10^3^
	Median	1.50 × 10^3^	2.74 × 10^3^	3.31 × 10^3^	8.74 × 10^3^	7.71 × 10^3^	1.72 × 10^3^	2.20 × 10^4^	8.89 × 10^3^	4.06 × 10^3^
	Rank	1	3	6	8	5	2	9	7	4
C17-F16	Mean	1.60 × 10^3^	1.63 × 10^3^	1.85 × 10^3^	1.78 × 10^3^	1.73 × 10^3^	1.67 × 10^3^	2.09 × 10^3^	1.91 × 10^3^	1.80 × 10^3^
	Best	1.60 × 10^3^	1.61 × 10^3^	1.68 × 10^3^	1.64 × 10^3^	1.65 × 10^3^	1.65 × 10^3^	1.94 × 10^3^	1.81 × 10^3^	1.71 × 10^3^
	Worst	1.60 × 10^3^	1.65 × 10^3^	2.12 × 10^3^	1.88 × 10^3^	1.84 × 10^3^	1.73 × 10^3^	2.20 × 10^3^	2.07 × 10^3^	1.83 × 10^3^
	Std	5.59 × 10^−1^	1.69 × 10^1^	2.01 × 10^2^	1.13 × 10^2^	8.59 × 10^1^	3.61 × 10^1^	1.08 × 10^2^	1.17 × 10^2^	5.38 × 10^1^
	Median	1.60 × 10^3^	1.62 × 10^3^	1.81 × 10^3^	1.80 × 10^3^	1.71 × 10^3^	1.66 × 10^3^	2.11 × 10^3^	1.88 × 10^3^	1.82 × 10^3^
	Rank	1	2	7	5	4	3	9	8	6
C17-F17	Mean	1.72 × 10^3^	1.73 × 10^3^	1.85 × 10^3^	1.83 × 10^3^	1.74 × 10^3^	1.76 × 10^3^	1.82 × 10^3^	1.75 × 10^3^	1.76 × 10^3^
	Best	1.72 × 10^3^	1.73 × 10^3^	1.76 × 10^3^	1.76 × 10^3^	1.73 × 10^3^	1.75 × 10^3^	1.75 × 10^3^	1.75 × 10^3^	1.75 × 10^3^
	Worst	1.73 × 10^3^	1.73 × 10^3^	1.98 × 10^3^	1.89 × 10^3^	1.75 × 10^3^	1.77 × 10^3^	2.01 × 10^3^	1.76 × 10^3^	1.76 × 10^3^
	Std	2.68 × 10^0^	2.25 × 10^0^	9.75 × 10^1^	6.26 × 10^1^	8.94 × 10^0^	9.67 × 10^0^	1.26 × 10^2^	5.64 × 10^0^	2.38 × 10^0^
	Median	1.72 × 10^3^	1.73 × 10^3^	1.84 × 10^3^	1.84 × 10^3^	1.74 × 10^3^	1.76 × 10^3^	1.76 × 10^3^	1.75 × 10^3^	1.76 × 10^3^
	Rank	1	2	9	8	3	6	7	4	5
C17-F18	Mean	1.80 × 10^3^	6.51 × 10^3^	2.04 × 10^4^	1.07 × 10^4^	2.54 × 10^4^	2.85 × 10^4^	6.19 × 10^3^	2.11 × 10^4^	1.24 × 10^4^
	Best	1.80 × 10^3^	2.61 × 10^3^	6.90 × 10^3^	4.49 × 10^3^	5.83 × 10^3^	2.32 × 10^4^	2.66 × 10^3^	2.84 × 10^3^	3.37 × 10^3^
	Worst	1.80 × 10^3^	9.27 × 10^3^	3.45 × 10^4^	1.66 × 10^4^	3.92 × 10^4^	3.56 × 10^4^	1.06 × 10^4^	3.93 × 10^4^	1.79 × 10^4^
	Std	4.25 × 10^−1^	2.89 × 10^3^	1.51 × 10^4^	5.33 × 10^3^	1.45 × 10^4^	5.72 × 10^3^	3.31 × 10^3^	1.88 × 10^4^	6.33 × 10^3^
	Median	1.80 × 10^3^	7.09 × 10^3^	2.01 × 10^4^	1.09 × 10^4^	2.82 × 10^4^	2.76 × 10^4^	5.75 × 10^3^	2.12 × 10^4^	1.42 × 10^4^
	Rank	1	3	6	4	8	9	2	7	5
C17-F19	Mean	1.90 × 10^3^	3.32 × 10^3^	6.21 × 10^4^	8.14 × 10^4^	9.01 × 10^3^	4.59 × 10^3^	3.31 × 10^4^	2.41 × 10^4^	6.02 × 10^3^
	Best	1.90 × 10^3^	1.91 × 10^3^	1.96 × 10^3^	2.36 × 10^3^	1.93 × 10^3^	2.04 × 10^3^	1.76 × 10^4^	2.60 × 10^3^	2.20 × 10^3^
	Worst	1.90 × 10^3^	4.22 × 10^3^	2.40 × 10^5^	2.69 × 10^5^	1.35 × 10^4^	1.21 × 10^4^	5.01 × 10^4^	7.41 × 10^4^	9.58 × 10^3^
	Std	4.70 × 10^−1^	1.04 × 10^3^	1.18 × 10^5^	1.26 × 10^5^	5.19 × 10^3^	5.00 × 10^3^	1.50 × 10^4^	3.37 × 10^4^	3.05 × 10^3^
	Median	1.90 × 10^3^	3.58 × 10^3^	3.37 × 10^3^	2.70 × 10^4^	1.03 × 10^4^	2.12 × 10^3^	3.23 × 10^4^	9.83 × 10^3^	6.15 × 10^3^
	Rank	1	2	8	9	5	3	7	6	4
C17-F20	Mean	2.01 × 10^3^	2.02 × 10^3^	2.30 × 10^3^	2.18 × 10^3^	2.06 × 10^3^	2.07 × 10^3^	2.25 × 10^3^	2.16 × 10^3^	2.05 × 10^3^
	Best	2.00 × 10^3^	2.01 × 10^3^	2.20 × 10^3^	2.17 × 10^3^	2.04 × 10^3^	2.06 × 10^3^	2.17 × 10^3^	2.14 × 10^3^	2.03 × 10^3^
	Worst	2.02 × 10^3^	2.04 × 10^3^	2.45 × 10^3^	2.20 × 10^3^	2.09 × 10^3^	2.08 × 10^3^	2.36 × 10^3^	2.19 × 10^3^	2.06 × 10^3^
	Std	1.06 × 10^1^	1.23 × 10^1^	1.17 × 10^2^	1.25 × 10^1^	2.45 × 10^1^	8.58 × 10^0^	8.92 × 10^1^	2.68 × 10^1^	1.05 × 10^1^
	Median	2.01 × 10^3^	2.02 × 10^3^	2.27 × 10^3^	2.18 × 10^3^	2.05 × 10^3^	2.07 × 10^3^	2.23 × 10^3^	2.16 × 10^3^	2.05 × 10^3^
	Rank	1	2	9	7	4	5	8	6	3
C17-F21	Mean	2.23 × 10^3^	2.24 × 10^3^	2.29 × 10^3^	2.29 × 10^3^	2.31 × 10^3^	2.30 × 10^3^	2.35 × 10^3^	2.32 × 10^3^	2.30 × 10^3^
	Best	2.20 × 10^3^	2.22 × 10^3^	2.21 × 10^3^	2.23 × 10^3^	2.29 × 10^3^	2.21 × 10^3^	2.34 × 10^3^	2.31 × 10^3^	2.23 × 10^3^
	Worst	2.31 × 10^3^	2.31 × 10^3^	2.38 × 10^3^	2.34 × 10^3^	2.32 × 10^3^	2.33 × 10^3^	2.36 × 10^3^	2.33 × 10^3^	2.33 × 10^3^
	Std	5.45 × 10^1^	4.50 × 10^1^	9.54 × 10^1^	5.94 × 10^1^	9.87 × 10^0^	5.65 × 10^1^	7.09 × 10^0^	1.18 × 10^1^	4.82 × 10^1^
	Median	2.20 × 10^3^	2.22 × 10^3^	2.30 × 10^3^	2.29 × 10^3^	2.31 × 10^3^	2.32 × 10^3^	2.35 × 10^3^	2.31 × 10^3^	2.32 × 10^3^
	Rank	1	2	4	3	7	6	9	8	5
C17-F22	Mean	2.28 × 10^3^	2.29 × 10^3^	2.65 × 10^3^	2.32 × 10^3^	2.31 × 10^3^	2.32 × 10^3^	2.30 × 10^3^	2.31 × 10^3^	2.32 × 10^3^
	Best	2.23 × 10^3^	2.24 × 10^3^	2.25 × 10^3^	2.30 × 10^3^	2.29 × 10^3^	2.31 × 10^3^	2.29 × 10^3^	2.30 × 10^3^	2.31 × 10^3^
	Worst	2.30 × 10^3^	2.30 × 10^3^	3.06 × 10^3^	2.32 × 10^3^	2.32 × 10^3^	2.32 × 10^3^	2.30 × 10^3^	2.34 × 10^3^	2.32 × 10^3^
	Std	3.77 × 10^1^	3.20 × 10^1^	3.93 × 10^2^	8.90 × 10^0^	1.25 × 10^1^	4.46 × 10^0^	3.86 × 10^0^	2.22 × 10^1^	5.15 × 10^0^
	Median	2.30 × 10^3^	2.30 × 10^3^	2.65 × 10^3^	2.32 × 10^3^	2.31 × 10^3^	2.32 × 10^3^	2.30 × 10^3^	2.30 × 10^3^	2.32 × 10^3^
	Rank	1	2	9	7	4	8	3	5	6
C17-F23	Mean	2.61 × 10^3^	2.61 × 10^3^	2.67 × 10^3^	2.66 × 10^3^	2.62 × 10^3^	2.64 × 10^3^	2.74 × 10^3^	2.64 × 10^3^	2.66 × 10^3^
	Best	2.61 × 10^3^	2.61 × 10^3^	2.63 × 10^3^	2.65 × 10^3^	2.61 × 10^3^	2.63 × 10^3^	2.68 × 10^3^	2.64 × 10^3^	2.64 × 10^3^
	Worst	2.62 × 10^3^	2.62 × 10^3^	2.71 × 10^3^	2.68 × 10^3^	2.63 × 10^3^	2.65 × 10^3^	2.89 × 10^3^	2.66 × 10^3^	2.66 × 10^3^
	Std	3.65 × 10^0^	3.25 × 10^0^	3.63 × 10^1^	1.37 × 10^1^	7.45 × 10^0^	8.49 × 10^0^	9.85 × 10^1^	8.79 × 10^0^	1.32 × 10^1^
	Median	2.61 × 10^3^	2.61 × 10^3^	2.66 × 10^3^	2.66 × 10^3^	2.62 × 10^3^	2.64 × 10^3^	2.70 × 10^3^	2.64 × 10^3^	2.66 × 10^3^
	Rank	1	2	8	7	3	4	9	5	6
C17-F24	Mean	2.50 × 10^3^	2.55 × 10^3^	2.75 × 10^3^	2.77 × 10^3^	2.73 × 10^3^	2.74 × 10^3^	2.57 × 10^3^	2.75 × 10^3^	2.71 × 10^3^
	Best	2.50 × 10^3^	2.54 × 10^3^	2.63 × 10^3^	2.74 × 10^3^	2.71 × 10^3^	2.73 × 10^3^	2.50 × 10^3^	2.73 × 10^3^	2.52 × 10^3^
	Worst	2.50 × 10^3^	2.55 × 10^3^	2.83 × 10^3^	2.80 × 10^3^	2.75 × 10^3^	2.74 × 10^3^	2.80 × 10^3^	2.76 × 10^3^	2.78 × 10^3^
	Std	2.08 × 10^−4^	3.46 × 10^0^	8.73 × 10^1^	2.29 × 10^1^	1.73 × 10^1^	3.17 × 10^0^	1.48 × 10^2^	1.26 × 10^1^	1.24 × 10^2^
	Median	2.50 × 10^3^	2.54 × 10^3^	2.77 × 10^3^	2.77 × 10^3^	2.72 × 10^3^	2.74 × 10^3^	2.50 × 10^3^	2.75 × 10^3^	2.76 × 10^3^
	Rank	1	2	8	9	5	6	3	7	4
C17-F25	Mean	2.90 × 10^3^	2.91 × 10^3^	3.04 × 10^3^	2.90 × 10^3^	2.93 × 10^3^	2.93 × 10^3^	2.93 × 10^3^	2.92 × 10^3^	2.95 × 10^3^
	Best	2.90 × 10^3^	2.90 × 10^3^	2.94 × 10^3^	2.77 × 10^3^	2.91 × 10^3^	2.91 × 10^3^	2.90 × 10^3^	2.90 × 10^3^	2.94 × 10^3^
	Worst	2.90 × 10^3^	2.91 × 10^3^	3.27 × 10^3^	2.95 × 10^3^	2.94 × 10^3^	2.95 × 10^3^	2.94 × 10^3^	2.94 × 10^3^	2.96 × 10^3^
	Std	3.36 × 10^−8^	2.98 × 10^0^	1.54 × 10^2^	8.55 × 10^1^	1.49 × 10^1^	1.86 × 10^1^	2.04 × 10^1^	2.43 × 10^1^	8.80 × 10^0^
	Median	2.90 × 10^3^	2.91 × 10^3^	2.97 × 10^3^	2.93 × 10^3^	2.94 × 10^3^	2.93 × 10^3^	2.94 × 10^3^	2.92 × 10^3^	2.95 × 10^3^
	Rank	2	3	9	1	7	6	5	4	8
C17-F26	Mean	2.83 × 10^3^	2.89 × 10^3^	3.76 × 10^3^	3.92 × 10^3^	3.13 × 10^3^	3.19 × 10^3^	3.15 × 10^3^	2.90 × 10^3^	2.89 × 10^3^
	Best	2.80 × 10^3^	2.82 × 10^3^	3.41 × 10^3^	3.09 × 10^3^	2.89 × 10^3^	2.90 × 10^3^	2.80 × 10^3^	2.80 × 10^3^	2.71 × 10^3^
	Worst	2.90 × 10^3^	2.98 × 10^3^	4.11 × 10^3^	4.50 × 10^3^	3.70 × 10^3^	3.83 × 10^3^	4.21 × 10^3^	3.01 × 10^3^	3.09 × 10^3^
	Std	5.00 × 10^1^	7.49 × 10^1^	3.82 × 10^2^	6.03 × 10^2^	3.80 × 10^2^	4.32 × 10^2^	7.04 × 10^2^	8.42 × 10^1^	1.93 × 10^2^
	Median	2.80 × 10^3^	2.87 × 10^3^	3.76 × 10^3^	4.04 × 10^3^	2.97 × 10^3^	3.01 × 10^3^	2.80 × 10^3^	2.89 × 10^3^	2.88 × 10^3^
	Rank	1	2	8	9	5	7	6	4	3
C17-F27	Mean	3.09 × 10^3^	3.09 × 10^3^	3.17 × 10^3^	3.13 × 10^3^	3.10 × 10^3^	3.11 × 10^3^	3.24 × 10^3^	3.13 × 10^3^	3.16 × 10^3^
	Best	3.09 × 10^3^	3.09 × 10^3^	3.15 × 10^3^	3.09 × 10^3^	3.09 × 10^3^	3.10 × 10^3^	3.23 × 10^3^	3.10 × 10^3^	3.12 × 10^3^
	Worst	3.09 × 10^3^	3.09 × 10^3^	3.20 × 10^3^	3.23 × 10^3^	3.10 × 10^3^	3.17 × 10^3^	3.24 × 10^3^	3.18 × 10^3^	3.21 × 10^3^
	Std	3.66 × 10^−1^	7.67 × 10^−1^	2.51 × 10^1^	6.45 × 10^1^	4.40 × 10^0^	3.62 × 10^1^	7.38 × 10^0^	3.50 × 10^1^	4.07 × 10^1^
	Median	3.09 × 10^3^	3.09 × 10^3^	3.17 × 10^3^	3.11 × 10^3^	3.09 × 10^3^	3.10 × 10^3^	3.24 × 10^3^	3.13 × 10^3^	3.15 × 10^3^
	Rank	1	2	8	5	3	4	9	6	7
C17-F28	Mean	3.10 × 10^3^	3.16 × 10^3^	3.42 × 10^3^	3.34 × 10^3^	3.38 × 10^3^	3.32 × 10^3^	3.44 × 10^3^	3.30 × 10^3^	3.24 × 10^3^
	Best	3.10 × 10^3^	3.15 × 10^3^	3.21 × 10^3^	3.17 × 10^3^	3.35 × 10^3^	3.21 × 10^3^	3.38 × 10^3^	3.17 × 10^3^	3.14 × 10^3^
	Worst	3.10 × 10^3^	3.16 × 10^3^	3.60 × 10^3^	3.44 × 10^3^	3.40 × 10^3^	3.38 × 10^3^	3.47 × 10^3^	3.38 × 10^3^	3.50 × 10^3^
	Std	7.84 × 10^−5^	3.72 × 10^0^	1.60 × 10^2^	1.19 × 10^2^	1.86 × 10^1^	8.13 × 10^1^	3.82 × 10^1^	9.33 × 10^1^	1.72 × 10^2^
	Median	3.10 × 10^3^	3.16 × 10^3^	3.43 × 10^3^	3.38 × 10^3^	3.38 × 10^3^	3.34 × 10^3^	3.45 × 10^3^	3.32 × 10^3^	3.16 × 10^3^
	Rank	1	2	8	6	7	5	9	4	3
C17-F29	Mean	3.15 × 10^3^	3.15 × 10^3^	3.29 × 10^3^	3.35 × 10^3^	3.17 × 10^3^	3.21 × 10^3^	3.31 × 10^3^	3.26 × 10^3^	3.23 × 10^3^
	Best	3.14 × 10^3^	3.14 × 10^3^	3.21 × 10^3^	3.25 × 10^3^	3.16 × 10^3^	3.17 × 10^3^	3.23 × 10^3^	3.17 × 10^3^	3.19 × 10^3^
	Worst	3.15 × 10^3^	3.16 × 10^3^	3.43 × 10^3^	3.47 × 10^3^	3.19 × 10^3^	3.23 × 10^3^	3.49 × 10^3^	3.34 × 10^3^	3.28 × 10^3^
	Std	8.92 × 10^0^	7.63 × 10^0^	9.77 × 10^1^	9.33 × 10^1^	1.35 × 10^1^	3.19 × 10^1^	1.18 × 10^2^	7.85 × 10^1^	4.06 × 10^1^
	Median	3.15 × 10^3^	3.15 × 10^3^	3.26 × 10^3^	3.33 × 10^3^	3.17 × 10^3^	3.22 × 10^3^	3.26 × 10^3^	3.27 × 10^3^	3.23 × 10^3^
	Rank	1	2	7	9	3	4	8	6	5
C17-F30	Mean	3.40 × 10^3^	1.43 × 10^5^	7.22 × 10^5^	1.14 × 10^6^	6.99 × 10^5^	5.84 × 10^4^	8.31 × 10^5^	3.72 × 10^5^	1.47 × 10^6^
	Best	3.40 × 10^3^	3.92 × 10^3^	1.84 × 10^4^	2.05 × 10^5^	5.99 × 10^3^	2.83 × 10^4^	5.13 × 10^5^	6.27 × 10^3^	5.05 × 10^5^
	Worst	3.41 × 10^3^	5.20 × 10^5^	1.51 × 10^6^	2.71 × 10^6^	2.58 × 10^6^	9.79 × 10^4^	1.14 × 10^6^	7.38 × 10^5^	3.34 × 10^6^
	Std	4.75 × 10^0^	2.52 × 10^5^	8.10 × 10^5^	1.17 × 10^6^	1.26 × 10^6^	3.40 × 10^4^	2.57 × 10^5^	4.22 × 10^5^	1.34 × 10^6^
	Median	3.40 × 10^3^	2.33 × 10^4^	6.81 × 10^5^	8.22 × 10^5^	1.03 × 10^5^	5.37 × 10^4^	8.34 × 10^5^	3.72 × 10^5^	1.01 × 10^6^
	Rank	1	3	6	8	5	2	7	4	9
Sum rank		33	70	217	200	137	158	175	148	167
Mean rank		1.14 × 10^0^	2.41 × 10^0^	7.48 × 10^0^	6.90 × 10^0^	4.72 × 10^0^	5.45 × 10^0^	6.03 × 10^0^	5.10 × 10^0^	5.76 × 10^0^
Total rank		1	2	9	8	3	5	7	4	6

**Table 9:** Wilcoxon rank sum test results for the indicated algorithm and function.

Compared Algorithm	Objective Function Type			
	Unimodal	High−Dimensional	Fixed−Dimensional	CEC 2017
OOBO vs. MPA	1.01 × 10^−24^	6.98 × 10^−15^	1.02 × 10^−8^	1.22 × 10^−18^
OOBO vs. TSA	1.01 × 10^−24^	1.28 × 10^−19^	1.44 × 10^−34^	2.41 × 10^−21^
OOBO vs. WOA	1.01 × 10^−24^	5.16 × 10^−14^	1.44 × 10^−34^	5.93 × 10^−21^
OOBO vs. GWO	1.01 × 10^−24^	7.58 × 10^−16^	1.44 × 10^−34^	1.97 × 10^−21^
OOBO vs. TLBO	1.01 × 10^−24^	1.04 × 10^−14^	1.44 × 10^−34^	7.05 × 10^−21^
OOBO vs. GSA	1.01 × 10^−24^	1.97 × 10^−21^	1.46 × 10^−13^	2.13 × 10^−21^
OOBO vs. PSO	1.01 × 10^−24^	1.97 × 10^−21^	1.2 × 10^−16^	1.97 × 10^−21^
OOBO vs. GA	1.01 × 10^−24^	1.97 × 10^−21^	1.44 × 10^−34^	2.09 × 10^−20^

**Table 10:** Performance of the indicated algorithm on the pressure vessel design problem.

Algorithm	Optimum Cost	Optimum Variables			
		$T_{s}$	$T_{h}$	R	L
MPA	5885.577	0.778210	0.384889	40.31504	200
TSA	5880.070	0.778099	0.383241	40.31512	200
WOA	6137.372	0.817577	0.417932	41.74939	183.5727
GWO	5889.369	0.779035	0.384660	40.32779	199.6503
TLBO	6011.515	0.845719	0.418564	43.81627	156.3816
GSA	11550.30	1.085800	0.949614	49.34523	169.4874
PSO	5891.388	0.778961	0.384683	40.32091	200
GA	5890.328	0.752362	0.399540	40.45251	198.0027
OOBO	5870.846	0.778080	0.383210	40.31502	200

**Table 11:** Statistical results of the indicated algorithm on the pressure vessel design problem.

Statistical Indicator	Algorithm								
	MPA	TSA	WOA	GWO	TLBO	GSA	PSO	GA	OOBO
Best	5885.577	5880.07	6137.372	5889.369	6011.515	11550.3	5891.388	5890.328	5870.846
Mean	5887.444	5884.14	6326.761	5891.525	6477.305	23342.29	6531.503	6264.005	5880.524
Worst	5892.321	5891.31	6512.354	5894.624	7250.917	33226.25	7394.588	7005.75	5882.658
Std	2.893	24.341	126.609	13.91	327.007	5790.625	534.119	496.128	9.125
Median	5886.228	5883.515	6318.318	5890.65	6397.481	24010.04	6416.114	6112.69	5875.969

**Table 12:** Performance of the indicated algorithm on the speed reducer design problem.

Algorithm	Optimum Cost	Optimum Variables						
		$x_{1}$	$x_{2}$	$x_{3}$	$x_{4}$	$x_{5}$	$x_{6}$	$x_{7}$
MPA	2999.446	3.49160	0.7	17	7.3	7.8	3.34938	5.288470
TSA	2994.247	3.50123	0.7	17	7.3	7.8	3.33421	5.265360
WOA	3030.563	3.50875	0.7	17	7.3	7.8	3.46102	5.289213
GWO	3002.316	3.50701	0.7	17	7.3798	7.79703	3.36211	5.302672
TLBO	3001.120	3.50214	0.7	17	7.3	7.8	3.29510	5.300210
GSA	3052.621	3.58612	0.7	17	8.3	7.8	3.37065	5.292941
PSO	3005.763	3.50023	0.7	17	8.3	7.8	3.35241	5.286715
GA	3068.128	3.51025	0.7	17	8.34821	7.8	3.35036	5.302641
OOBO	2989.852	3.50120	0.7	17	7.3	7.8	3.33412	5.265310

**Table 13:** Statistical results of the indicated algorithm on the speed reducer design problem.

Statistical Indicator	Algorithm								
	MPA	TSA	WOA	GWO	TLBO	GSA	PSO	GA	OOBO
Best	2999.446	2994.247	3030.563	3002.316	3001.120	3052.621	3005.763	3068.128	2989.852
Mean	2999.640	2997.482	3065.917	3005.845	3028.841	3170.334	3105.252	3186.523	2993.010
Worst	3003.889	2999.092	3104.779	3008.752	3060.958	3363.873	3211.174	3313.199	2998.425
Std	1.9319	1.7809	18.074	5.8379	13.0186	92.5726	79.6381	17.1186	1.2241
Median	2999.187	2996.318	3065.609	3004.519	3027.031	3156.752	3105.252	3198.187	2992.018

**Table 14:** Performance of the indicated algorithm on the welded beam design problem.

Algorithm	Optimum Cost	Optimum Variables			
		h	l	t	b
MPA	1.725834	0.205584	3.475193	9.036703	0.205832
TSA	1.723761	0.205432	3.472688	9.036119	0.201173
WOA	1.759349	0.204715	3.536645	9.005190	0.210046
GWO	1.727168	0.205699	3.475751	9.037868	0.206250
TLBO	1.725645	0.205632	3.472450	9.041835	0.205730
GSA	2.173075	0.147113	5.491293	10.00	0.217747
PSO	1.820577	0.197431	3.315393	10.00	0.201415
GA	1.874158	0.164187	4.032944	10.00	0.223669
OOBO	1.720985	0.203280	3.471150	9.0350	0.201160

**Table 15:** Statistical results of the indicated algorithm on the welded beam design problem.

Statistical Indicator	Algorithm								
	MPA	TSA	WOA	GWO	TLBO	GSA	PSO	GA	OOBO
Best	1.725834	1.723761	1.759349	1.727168	1.725645	2.173075	1.820577	1.874158	1.720985
Mean	1.726001	1.725297	1.817839	1.727301	1.729853	2.544493	2.230533	2.119452	1.725021
Worst	1.726237	1.727384	1.873595	1.727737	1.741825	3.003957	3.048536	2.320357	1.727205
Std	0.000287	0.004325	0.027546	0.001157	0.004866	0.255885	0.324557	0.034823	0.003316
Median	1.725960	1.724571	1.820310	1.727260	1.727593	2.495364	2.244887	2.097258	1.724224

**Table 16:** Performance of the indicated algorithm on the tension spring design problem.

Algorithm	Optimum Cost	Optimum Variables		
		d	D	P
MPA	0.012675	0.051149	0.343785	12.09671
TSA	0.012658	0.051092	0.342942	12.09101
WOA	0.012711	0.050785	0.334812	12.72396
GWO	0.012679	0.050183	0.341575	12.07470
TLBO	0.012818	0.050012	0.315988	14.22765
GSA	0.012875	0.050005	0.317344	14.23009
PSO	0.013194	0.050000	0.310445	15.00150
GA	0.013037	0.050105	0.310142	14.00140
OOBO	0.012655	0.051070	0.342880	12.08809

**Table 17:** Statistical results of the indicated algorithm on the tension spring design problem.

Statistical Indicator	Algorithm								
	MPA	TSA	WOA	GWO	TLBO	GSA	PSO	GA	OOBO
Best	0.012675	0.01266	0.012711	0.012679	0.012818	0.012875	0.013194	0.013037	0.012655
Mean	0.012685	0.01268	0.012841	0.012698	0.014465	0.013440	0.014818	0.014037	0.012678
Worst	0.012716	0.01267	0.012999	0.012722	0.017842	0.014213	0.017865	0.016253	0.012668
Std	2.70E-05	0.00102	7.80E-05	4.10E-05	0.001622	0.000287	0.002272	0.002073	0.001010
Median	0.012688	0.01268	0.012846	0.012701	0.014022	0.013369	0.013194	0.013003	0.012676

## Data Availability

Not applicable.
